# Methods for *in vitro* CRISPR/CasRx-Mediated RNA Editing

**DOI:** 10.3389/fcell.2021.667879

**Published:** 2021-06-11

**Authors:** Yu-Fan Chuang, Peng-Yuan Wang, Satheesh Kumar, Suraj Lama, Fan-Li Lin, Guei-Sheung Liu

**Affiliations:** ^1^Shenzhen Key Laboratory of Biomimetic Materials and Cellular Immunomodulation, Shenzhen Institute of Advanced Technology, Chinese Academy of Sciences, Shenzhen, China; ^2^Menzies Institute for Medical Research, University of Tasmania, Hobart, TAS, Australia; ^3^Ophthalmology, Department of Surgery, University of Melbourne, East Melbourne, VIC, Australia; ^4^Aier Eye Institute, Changsha, China

**Keywords:** CRISPR/Cas13d, CasRx, RNA editing, VEGF (Vascular endothelial growth factor), AAV (Adeno-associated virus)

## Abstract

Specific changes in the genome have been accomplished by the revolutionary gene-editing tool known as clustered regularly interspaced short palindromic repeats (CRISPR)/CRISPR-associated (Cas) system. The advent of programmable RNA editing CRISPR/Cas nucleases has made this gene-editing tool safer and more precise. Specifically, CasRx, a family member of the Cas13d family, has shown great therapeutic potential. Here, we describe the *in vitro* methods of utilizing this powerful RNA editing platform and determine the RNA editing efficiencies for CasRx with different forms of guide RNAs (also known as gRNA or sgRNA).

## Highlights

– Detailed step-by-step methods for *in vitro* CRISPR/CasRx system targeting *VEGFA* mRNA.
– Comparison of the RNA editing efficiency between different forms of sgRNA.
– Generation of AAV all-in-one vector consisting of up to three pre-sgRNAs for effective knockdown of *VEGFA* gene expression.

## Introduction

The discovery of the clustered regularly interspaced short palindromic repeats (CRISPR)/CRISPR-associated (Cas) system has provided the opportunity for scientists to make precise changes in the genome. CRISPR/Cas-type II RNA-guided nucleases, including *Streptococcus pyogenes* Cas9 (SpCas9), have been extensively explored and demonstrated to possess excellent DNA editing capabilities (Jinek et al., 2012). However, the large size of SpCas9 makes packaging into low-capacity vectors such as adeno-associated virus (AAV) for *in vivo* delivery or other therapeutic applications challenging. This warrants the exploitation of smaller-sized enzymes.

Unlike DNA manipulation using the CRISPR/Cas system, there is a significant lack of literature for CRISPR/Cas-based RNA modification. Recently, scientists characterized Cas13 enzymes and demonstrated programmable RNA editing superior in efficiency and specificity compared to existing RNA-targeting approaches (Abudayyeh et al., 2017; Cox et al., 2017; Liu et al., 2017; Konermann et al., 2018). CRISPR/Cas13 was also reported safer than existing CRISPR/Cas systems due to the lack of genomic alterations. In 2018, the smallest of RNA-targeting Cas nucleases, Cas13d, was described. Within the Cas13d family, CasRx (also known as RfxCas13d), from *Ruminococcus flavifaciens*, possesses the highest RNA cleavage activity and specificity in human cells. RNA targeting by CasRx also fared better than short hairpin RNA (shRNA) interference. Importantly, Cas13d nucleases can process CRISPR arrays, allowing for multiplex targeting (Konermann et al., 2018). Subsequent studies with CasRx have shown efficient messenger RNA (mRNA) knockdown in various animal models and transgenic expression in plants (Mahas et al., 2019; Kushawah et al., 2020). Notably, the therapeutic potential of CRISPR/CasRx was demonstrated in mouse models of neovascular age-related macular degeneration (nAMD) using AAV vectors. Delivery of the CRISPR/CasRx system successfully suppressed the mRNA of vascular endothelial growth factor (VEGF), the key factor for pathogenic ocular angiogenesis, and also subsequently showed a reduction in the area of choroidal neovascularization (CNV), the hallmark of nAMD (Zhou et al., 2020). These studies indicate the therapeutic potential for the CRISPR/CasRx system.

Here, we describe three different methods to perform efficient CRISPR/CasRx-mediated RNA knockdown of vascular endothelial growth factor A (VEGFA). We examined the RNA knockdown efficiencies of the CRISPR/CasRx system using different forms of single guide RNA (sgRNA) by targeting *VEGFA* mRNA. For application in development of therapeutics, we generated all-in-one AAV constructs consisting of CasRx and a single pre-sgRNA or multiple pre-sgRNAs (array) to examine the RNA knockdown efficiency of the system. An instructional manual for RNA editing *in vitro* with CasRx using variants of guide RNAs is described.

## Materials

### Mammalian Cells

• Human embryonic kidney (HEK) 293FT cell line (cat. R70007; Life Technologies Australia, Mulgrave, VIC, Australia)
• Human Müller cell line, Moorfields/Institute of Ophthalmology–Müller 1 (MIO-M1), which was obtained from the UCL Institute of Ophthalmology, London (Limb et al., 2002).

### Gene Fragments

• LacZ sgRNA [Integrated DNA Technologies (IDT^TM^), Singapore]-gBlocks^TM^
• VEGFA sgRNA1 (IDT^TM^)-gBlocks^TM^
• VEGFA sgRNA2 (IDT^TM^)-gBlocks^TM^
• VEGFA sgRNA3 (IDT^TM^)-gBlocks^TM^
• VEGFA shRNA (IDT^TM^)-gBlocks^TM^
• VEGFA pre-sgRNA for Gibson assembly (IDT^TM^)-gBlocks^TM^
• VEGFA array pre-sgRNA for Gibson assembly (IDT^TM^)-gBlocks^TM^
• VEGFA shRNA for Gibson assembly (IDT^TM^)- gBlocks^TM^.

### Gene Fragment Amplification

• Primers (sgRNA cassettes forward primer and reverse primer; IDT^TM^) (see Supplementary Table 1)
• Tris-EDTA (TE) buffer, pH 8.0, RNase-free (cat. AM9849, Invitrogen, Scoresby, VIC, Australia)
• Q5^®^ High-Fidelity 2X Master Mix (cat. M0492S, New England Biolabs, Notting Hill, VIC, Australia)
• DNA Clean and Concentrator^TM^-25 (cat. D4034, Zymo Research, Chatswood, NSW, Australia).

### Plasmids

• EF1α-CasRx-2A-EGFP (pXR001; cat. 109049, Addgene)
• CasRx pre-gRNA cloning backbone (pXR004; cat. 109054, Addgene, Watertown, MA, USA)
• Control (non-targeting) pre-sgRNA (pM115; cat. 166867, Addgene)
• VEGFA pre-sgRNA (pM116; cat. 166868, Addgene)
• VEGFA pre-sgRNA array (pM117; cat. 166869, Addgene)
• VEGFA shRNA (pM106)
• pAAV-MCS Expression Vector (cat. VPK-410, Cell Biolabs, San Diego, CA, USA)
• pAAV-CasRx-control pre-sgRNA (cat. 166871, pM123; Addgene)
• pAAV-CasRx-VEGFA pre-sgRNA (pM124; cat. 166872, Addgene)
• pAAV-CasRx-VEGFA array pre-sgRNA (pM125; cat. 166873, Addgene).

### Oligonucleotide Annealing and Gibson Assembly

• Oligonucleotides (top and bottom; Integrated DNA Technologies)
• T4 polynucleotide kinase (PNK; cat. M0201S, New England BioLabs)
• Calf intestinal alkaline phosphatase (CIP; cat. F-201S, Thermo Fisher Scientific, Waltham, MA, USA)
• Adenosine 5′-Triphosphate (ATP) (cat. P0756S, New England Biolabs)
• T4 DNA ligase (cat. M0202T, New England Biolabs)
• Restriction enzymes: *Bbs*I, *Nhe*I, *Eco*RI, *Mlu*I, and *Rsr*II (New England BioLabs)
• NEBuilder HiFi DNA Gibson Assembly kit (cat. E2611S, New England BioLabs)
• Qubit dsDNA BR Assay kit (cat. Q32853, Invitrogen)
• Qubit Assay tubes (cat. Q32856, Invitrogen).

### Plasmid Amplification

• Luria broth base (Miller’s LB Broth Base)^TM^ (cat. 12795-027, Invitrogen)
• Luria agar base, Miller (cat. L2025, Sigma-Aldrich, North Ryde, NSW, Australia)
• Carbenicillin disodium salt (cat. C9231, Sigma-Aldrich)
• NEB^®^ Stable Competent *Escherichia coli* (high-efficiency) supplied with outgrowth medium (cat. C3040I, New England BioLabs)
• QIAprep Spin Miniprep kit (cat. 27106, QIAGEN, Chadstone, VIC, Australia)
• QIAGEN^®^ Plasmid Midi kit (cat. 12143, QIAGEN)
• QIAGEN^®^ Plasmid Maxi kit (cat. 12163, QIAGEN)
• PureLink^TM^ HiPure Precipitator Module (cat. K2100-21, Thermo Fisher Scientific).

### Plasmid Validation

• Restriction enzymes and buffers (New England BioLabs, see Supplementary Table 2)
• Agarose (cat. A9539, Sigma-Aldrich)
• 1-kb Plus DNA Ladder (cat. 10787018, Invitrogen)
• Gel loading dye, purple (6 × ) (cat. B7024S, New England BioLabs)
• Sequencing primer (LKO.1; IDT^TM^; see Supplementary Table 1).

### Cell Culture

• Dulbecco’s modified Eagle’s medium (DMEM) high glucose (cat. 11965092, Thermo Fisher Scientific)
• Antibiotic–antimycotic (cat. 15240096, Thermo Fisher Scientific)
• GlutaMAX^TM^ Supplement (cat. 35050061, Thermo Fisher Scientific)
• Fetal bovine serum (cat. F9423, Sigma-Aldrich)
• Falcon^®^ 75-cm^2^ rectangular canted neck cell culture flask with vented cap (cat. 353136, Falcon/Corning, Mulgrave, VIC, Australia)
• Multiwell cell culture plates (Falcon/Corning, Mulgrave, VIC, Australia)
• Dulbecco’s phosphate-buffered saline (DPBS), no calcium, no magnesium (cat. 14190144, Thermo Fisher Scientific)
• TryPLE^TM^ Express Enzyme (1 × ), phenol red (cat. 12605028, Thermo Fisher Scientific).

### Transfection and RNA Extraction

• DMEM, high glucose (cat. 11965092, Thermo Fisher Scientific)
• Opti-MEM^TM^ Reduced Serum Medium (cat. 31985070, Thermo Fisher Scientific)
• Lipofectamine^TM^ 2000 Transfection Reagent (cat. 11668019, Invitrogen)
• ViaFect^TM^ Transfection Reagent (cat. E4981, Promega, Alexandria, NSW, Australia)
• GENbag Anaer (cat. 45534, bioMérieux, Marcy-l’Étoile, France)
• Quick-RNA Miniprep kit (cat. R1055, Zymo Research).

### Reverse Transcription and Quantitative Polymerase Chain Reaction

• High-Capacity cDNA Reverse Transcription Kit (cat. 4368814, Applied Biosystems/Thermo Fisher Scientific)
• TaqMan^TM^ Fast Advanced Master Mix (cat. 4444557, Applied Biosystems/Thermo Fisher Scientific)
• VEGFA TaqMan^TM^ Fluorescent Real-Time PCR Primers (Hs00900055_m1, Applied Biosystems/Thermo Fisher Scientific)
• GAPDH TaqMan^TM^ Fluorescent Real-Time PCR Primers (Hs99999905_m1, Applied Biosystems/Thermo Fisher Scientific).

### Equipment

• Tissue culture equipment (e.g., cell incubators, cell counter, water baths, and microscopes)
• Centrifuges (e.g., high-speed centrifuge and microcentrifuge)
• Dry Block Heater (Thermo Fisher Scientific)
• NanoDrop 2000c (Thermo Fisher Scientific)
• Qubit 2.0 Fluorometer (Thermo Fisher Scientific)
• PCR thermal cycler (QuantStudio^TM^ 3 Flex Real-Time PCR System, Applied Biosystems/Thermo Fisher Scientific)
• Quantitative polymerase chain reaction (qPCR) instrument (VeritiPro Thermal Cycler, Thermo Fisher Scientific)
• Gel image system (AI600 Imager, GE Healthcare Life Sciences, Parramatta, NSW, Australia).

## Methods

### sgRNA Selection and Vector Design

**FIGURE 1:**
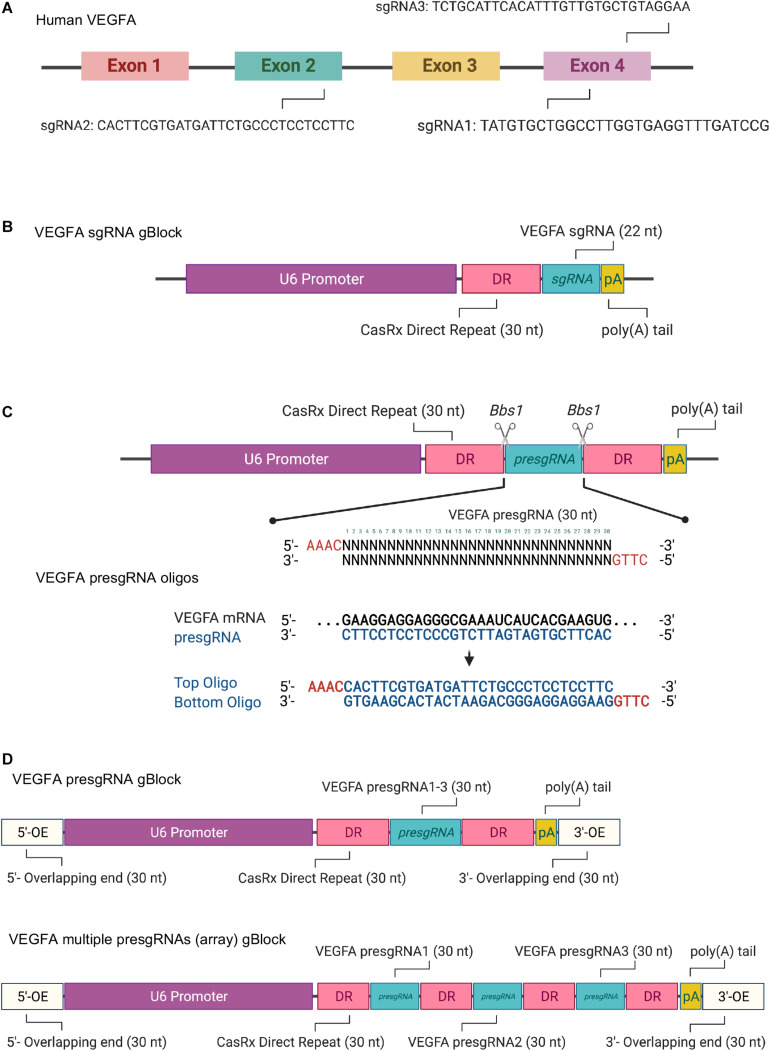
**(A)** Schematic illustration of the targeting sites of the selected single guide RNAs (sgRNAs) in human *VEGFA* mRNA. **(B)** Schematic illustration of the synthetic sgRNA expression cassette for CRISPR/CasRx-mediated RNA editing. **(C)** Schematic illustration of the design and pre-sgRNA cloning *via* oligonucleotide annealing. **(D)** Schematic illustration of the single pre-sgRNA and multiple pre-sgRNA (array) expression cassettes for Gibson assembly. Created with BioRender.com.

The transcript sequence of the target gene (human *VEGFA*) was obtained from NCBI^1^ (Figure 1A and Supplementary Figure 1). Two forms of CasRx sgRNAs, sgRNAs (22 nt) and pre-sgRNAs (30 nt), that do not target the stem–loop region of the target mRNA were selected for an increased targeting rate. The secondary structure of the target mRNA can be predicted using the RNAfold web server^2^ or other online tools. Since the selection parameters of the sgRNA target regions are similar to RNA interference, small interfering RNA (siRNA) designed web tools can be used for sgRNA selection. Three sgRNA/pre-sgRNAs targeting human *VEGFA* mRNA were designed for the study. The first VEGFA sgRNA/pre-sgRNA was designed based on the targeting sequence of bevasiranib, the first VEGFA-targeted siRNA to reach phase III clinical trial (Garba and Mousa, 2010). The other two sgRNA/pre-sgRNAs were manually designed to target the different regions in *VEGFA* mRNA, avoiding the stem–loop. In addition, short fragments of CasRx sgRNA expression cassette (gBlocks^TM^), CasRx pre-sgRNA expression plasmid, and all-in-one CasRx AAV plasmid were designed and illustrated using Benchling^3^.

It must be noted that the mRNA is single-stranded during the sgRNA design.

### CasRx sgRNA Expression Cassette and Amplification

CasRx sgRNAs (22 nt) targeting the three regions of the *VEGFA* mRNA were selected as described in section “sgRNA Selection and Vector Design.” The CasRx sgRNA expression cassettes contain an upstream U6 promoter, a 30-nt direct repeat (DR), 22-nt sgRNA (referred to as VEGFA sgRNA1, sgRNA2, or sgRNA3), and a poly(A) tail (Figure 1B and Supplementary Figure 2) synthesized as gBlocks^TM^ gene fragments. The CasRx sgRNA expression cassettes can be amplified by PCR with Q5^®^ High-Fidelity 2X master mix. The reaction solutions prepared are shown below.

**Table:** 

	Amount (μl)	Final concentration
Q5^®^ High-Fidelity 2X master mix	25	1 ×
sgRNA cassette forward (10 μM)	2.5	0.5 μM
sgRNA cassette reverse (10 μM)	2.5	0.5 μM
Template gBlocks^TM^	100 ng	< 1,000 ng
Nuclease-free water	Add up to 50	
Final volume	50	

The PCR thermal cycling conditions were as below.

**Table:** 

	Temperature (°C)	Time (s)
Initial denaturation	98	30
30 cycles	98	10
	66	30
	72	30
Final extension	72	120
Hold	4–10	

The PCR products were resolved by gel electrophoresis in 1% agarose gel to ensure correct size of the amplicon.

The amplified gene fragment was then purified using DNA Clean and Concentrator^TM^ Kit-25 (Zymo Research) and eluted in 10 mM Tris–HCl, pH 8 (or the elution buffer from the kit) (Supplementary Method—DNA Clean and Concentrator kit). DNA was quantified using Qubit^TM^ dsDNA BR Assay.

It should be noted that: (1) the annealing temperature is dependent on the GC content of the primers and (2) a 427-bp PCR band should be present on 1% agarose gel.

### CasRx Pre-sgRNA Vector Design and Construction

CasRx pre-sgRNAs (30 nt) targeting the three regions of the *VEGFA* mRNA were selected as described in section “sgRNA Selection and Vector Design.” The CasRx pre-sgRNAs for VEGFA (referred to as VEGFA pre-sgRNA1, pre-sgRNA2, and pre-sgRNA3) were cloned into the CasRx pre-gRNA cloning backbone plasmid (cat. 109054, Addgene) *via* oligonucleotide annealing or Gibson assembly methods.

#### Oligonucleotide Annealing

In order to clone the pre-sgRNAs into the CasRx pre-sgRNA cloning backbone, two oligonucleotides were designed and synthesized, as in Figure 1C and Supplementary Figure 3. The CasRx pre-sgRNA cloning backbone plasmid has the same overhangs after *Bbs*I digestion that allows the annealed oligonucleotides to be cloned into it.

##### Backbone plasmid digestion

Digest and dephosphorylate 2 μg of the CasRx pre-gRNA cloning backbone plasmid with *Bbs*I for 2 h at 37°C.

**Table:** 

Component	Amount/reaction (μl)
Plasmid (CasRx pre-sgRNA cloning backbone)	2 μg (depends on DNA concentration)
*Bbs*I	1 (20 U)
CutSmart^®^ buffer (10 × )	3
Nuclease-free water	Add up to 30
Final volume	30

After *Bbs*I digestion, 5′-phosphate groups were removed from the digested plasmid by calf intestinal alkaline phosphatase (CIP) to prevent self-ligation.

**Table:** 

Component	Amount/reaction (μl)
Digested plasmid mixture from the previous step	30
CIP	1 (1 U)
CutSmart^®^ buffer (10 × )	2
Nuclease-free water	Add up to 50
Final volume	50

DNA electrophoresis was performed in 0.8% agarose gel to ensure plasmid DNA was completely digested. The CIP-digested plasmid was then purified using QIAquick Gel Extraction kit (QIAGEN) and eluted in 10 mM Tris–HCl, pH 8 (or the elution buffer from kit) (Supplementary Method—QIAquick Gel Extraction kit).

It must be noted that an ∼3-kb digested band should be present on 0.8% agarose gel.

##### Oligonucleotide annealing reaction

Suspend the synthesized oligonucleotides (100 μM) and set up the annealing mixture.

**Table:** 

Component	Amount/reaction (μl)
Top oligonucleotides (100 μM)	1 (5 μM)
Bottom oligonucleotides (100 μM)	1 (5 μM)
ATP (25 mM) T4 polynucleotide kinase (PNK) PNK buffer (10 × )	2 (2.5 mM) 1 (10 U) 2
Nuclease-free water	13
Final volume	20

The annealing reaction was then performed with the thermal cycling conditions shown below.

**Table:** 

	Temperature (°C)	Time (min)
Step 1	37	60
Step 2	95	5
Step 3	4	Ramp by 0.1°C/s

##### DNA ligation

Dilute the annealed oligonucleotides (1:25) from the previous step. Set up the ligation reaction and incubate at 16°C overnight. Following incubation, store the samples on ice or at −20°C for subsequent transformation.

**Table:** 

Component	Amount/reaction (μl)
Linearized plasmid (CasRx pre-sgRNA cloning backbone)	50 ng (depends on DNA concentration)
Annealed oligonucleotides	1
T4 DNA ligase T4 DNA ligase buffer (10 × )	1 (20 U) 2
Nuclease-free water	Add up to 20
Final volume	20

It must be noted that a reaction without annealed oligonucleotides should be set up as a negative control.

#### Gibson Assembly

In order to clone the single pre-sgRNA or multiple pre-sgRNAs (array) into the desired vector, both insert DNA fragment (CasRx pre-sgRNA expression cassette) (Figure 1D and Supplementary Figures 4, 5) and linear plasmid (CasRx pre-sgRNA cloning backbone) can be assembled by Gibson assembly cloning. The CasRx pre-sgRNA expression cassette was synthesized as gBlocks^TM^ gene fragments, which contain a U6 promoter, DR, and pre-sgRNA, as well as with 30 bp at the left and right ends that overlapped with the linear plasmid sequences flanking the cleavage site. The CasRx pre-sgRNA expression cassette was ligated with the linearized plasmid *in vitro* using Gibson assembly reaction. Three CasRx pre-sgRNA plasmids (control pre-sgRNA, VEGFA pre-sgRNA, and VEGFA pre-sgRNA array) were generated for this study (Supplementary Figures 6–8).

##### Backbone vector digestion

Digest 2 μg of the CasRx pre-sgRNA cloning backbone plasmid with *Nhe*I and *Eco*RI overnight at 37°C.

**Table:** 

Component	Amount/reaction (μl)
Plasmid (CasRx pre-sgRNA cloning backbone)	2 μg (depends on DNA concentration)
*Nhe*I *Eco*RI	1 (20 U) 1 (20 U)
CutSmart^®^ buffer (10 × )	2
Nuclease-free water	Add up to 20
Final volume	20

DNA electrophoresis was performed in 0.8% agarose gel to ensure plasmid DNA was completely digested. The digested plasmid was then purified using QIAquick Gel Extraction kit (QIAGEN) and eluted using 10 mM Tris–HCl, pH 8 (or the elution buffer from the kit). DNA was quantified using Qubit dsDNA BR Assay.

Note that an ∼2.6-kb and a 359-bp digested band should be present on 0.8% agarose gel.

##### DNA fragment assembly reaction

Set up the DNA segments (linearized plasmid and synthesized U6-pre-sgRNA DNA fragment) for Gibson assembly reaction.

**Table:** 

Component	Amount/reaction (μl)
Linearized plasmid	0.02–0.5 pmol (depends on DNA concentration)
Pre-sgRNA expression cassette (gBlocks^TM^)	0.02–0.5 pmol (depends on DNA concentration)
Gibson assembly master mix (2 × )	10
Nuclease-free water	Add up to 20
Final volume	20

Incubate the assembly reaction in a thermal cycling condition at 50°C for 15 min. Following incubation, store the samples on ice or at −20°C for subsequent transformation.

The following must be noted: (1) For optimal cloning efficiency, use 50–100 ng of plasmid with a twofold molar excess of each insert. Use 5 × of insert if the size is less than 200 bp. Prolonged incubation up to 60 min may help improve assembly efficiency in some cases. (2) Avoid strong secondary structures in the homology region. Hairpins in this region can significantly reduce the efficiency of annealing two homologous ends.

### Transformation and Vector Validation

(i) Ten microliters of the ligation reaction or 2 μl of the assembly reaction was added into 50 μl of NEB Stable Competent *E. coli*. Eppendorf tubes were flicked five times to mix the cells with the plasmid DNA.
(ii) The mixture was placed on ice for 30 min and heat-shocked at 42°C for 30 s before being placed on ice for 5 min.
(iii) Of the NEB Stable outgrowth medium, 450 μl was added into the mixture and the tube was rotated at 37°C for 1 h.
(iv) One hundred microliters of the mixture was spread on carbenicillin (100 μg/ml)-containing agar plates. The plates were incubated overnight at optimal temperature (e.g., 37°C).
(v) A day after transformation, a single colony was selected and added to a tube containing 6 ml LB broth with carbenicillin (100 μg/ml) to generate miniculture. The mixture was shaken at a speed of 225 rpm for 16 h at optimal temperature (e.g., 37°C). The next day, miniprep was done using QIAprep Spin Miniprep kit (QIAGEN) and eluted in 10 mM Tris–HCl, pH 8 (or the elution buffer from the kit) (Supplementary Method—QIAprep Spin Miniprep kit).
(vii) For midiprep, 100 μl of the miniculture was added to 200 ml LB broth with carbenicillin (100 μg/ml), while for maxiprep, 1 ml of the miniculture was added to 500 ml LB broth with carbenicillin (100 μg/ml). The mixtures were shaken at 200 rpm for 16 h at optimal temperature (e.g., 37°C). Midiprep or maxiprep was performed the following day using QIAGEN^®^ Plasmid Midiprep kit or Maxiprep kit (QIAGEN) and eluted in 10 mM Tris–HCl, pH 8 (or the elution buffer from the kit) (Supplementary Method—QIAGEN^®^ Plasmid Midiprep kit).

Note that the liquid should be pushed out as slowly as possible to obtain a higher DNA concentration.

(viii) DNA was quantified using Nanodrop^TM^ before 500 ng of each plasmid DNA was used for validation. Preparation of the restriction enzyme digestion solution for validation is shown below.

**Table:** 

Component	Amount/reaction (μl)
DNA	Depends on DNA concentration
Restriction enzymes (depends on plasmid; see Supplementary Table 2)	0.5
Buffers (depends on restriction enzymes; see Supplementary Table 2)	2
Nuclease-free water	Add up to 20
Final volume	20

(ix) The restriction enzyme digestion solution was placed on a block heater at optimal temperature (e.g., 37°C) for at least 1 h. DNA electrophoresis was performed in 1% agarose gel to ensure digested bands matched the predicted bands. The validated plasmid was sequenced with LKO.1 primer (see Supplementary Table 1) to validate the insertion of CasRx pre-sgRNA.

Note that the heat shock temperature should be at exactly 42°C for 30 s.

For troubleshooting: (1) Formation of small colonies might be due to contamination or incorrect incubation temperature. (2) If the population of colonies is too high, decrease the amount of mixture spread on the agar plate or ensure that the antibiotics used are still efficient. If no colonies appear, ensure the correct antibiotic is used and competent cells are still viable. (3) Unclear digested bands might be due to incomplete digestion from inadequate incubation. Incubation on a block heater can be done overnight for complete digestion of plasmids.

### All-in-one AAV Vector Design and Construction

The all-in-one AAV construct consists of CasRx and single (or multiple) CasRx pre-sgRNA targeting *VEGFA* mRNA, which were based on the pAAV-MCS backbone plasmid (Cell Biolabs, Inc.) and generated *via* Gibson assembly. The all-in-one CasRx and pre-sgRNA expression cassette that contains a U6 promoter, DR, single pre-sgRNA (or multiple pre-sgRNAs), an elongation factor 1α short (EFS) promoter, and a CasRx-SV40 poly(A) signal was first synthesized as gBlocks^TM^ gene fragment. The pAAV-MCS backbone plasmid was linearized by *Mul*I/*Rsr*II digestion and a DNA fragment [CMV promoter–human b-globulin intron–hGH poly(A) signal] was removed at the same time. The expression cassette was then ligated with the linearized plasmid (pAAV-MCS) *in vitro* using Gibson assembly, as described in section “Gibson Assembly.” Three all-in-one pAAV plasmids (pAAV–CasRx–control pre-sgRNA, pAAV–CasRx–VEGFA pre-sgRNA, and pAAV–CasRx–VEGFA pre-sgRNA array) were generated for this study (Supplementary Figures 9–11).

### Cell Culture

HEK293FT cells were maintained in DMEM with 1% antibiotic–antimycotic, 1% GlutaMAX^TM^, and 10% FBS in a humidified 5% CO_2_ atmosphere at 37°C. HEK293FT cells were passaged every 3 days. The cells were rinsed with DPBS and passaged using TryPLE^TM^ for 3 min at 37°C. The cells were neutralized using a 1:3 ratio of TryPLE^TM^ to complete DMEM and subjected to cell counting. HEK293FT cells (5 × 10^5^) were seeded in a 75-cm^2^ cell culture flask for passaging. For experiments, HEK293FT cells were seeded into multiwell cell culture plates (six-well plate: 2 × 10^5^ cells/2 ml complete DMEM/well; 12-well plate: 10^5^ cells/1 ml complete DMEM/well) a day before transfection. Cell densities were at 50–70% on the day of transfection.

### Cell Transfection and RNA Extraction

#### Transfection of HEK293FT Cells

(i) The cells were seeded in multiwell cell culture plates a day before transfection, for the cell density to be at 50–70% during transfection.

For experiments using short fragments of the CasRx sgRNA expression cassette (gBlocks^TM^), prepare the Lipofectamine 2000/DNA mixture as follows:

**Table:** 

	Each well (six-well plate)	Each well (12-well plate)
CasRx plasmid DNA (ng)	1,600	800
VEGFA sgRNA gBlocks^TM^ (ng)	200	100
Lipofectamine 2000 (μl) [DNA (μg)/Lipofectamine 2000 (μl) = 1:3]	5.4	2.7
Opti-MEM (μl)	300	100

For experiments using dual plasmids (CasRx and pre-sgRNA expression vectors), prepare the Lipofectamine 2000/DNA mixture as follows:

**Table:** 

	Each well (six-well plate)	Each well (12-well plate)
CasRx plasmid (ng)	800	400
VEGFA pre-sgRNA plasmid (ng)	800	400
Lipofectamine 2000 (μl) [DNA (μg)/Lipofectamine 2000 (μl) = 1:3]	4.8	2.4
Opti-MEM (μl)	300	100

For experiments using the all-in-one pAAV–CasRx–pre-sgRNA plasmid, prepare the Lipofectamine 2000/DNA mixture as follows:

**Table:** 

	Each well (six-well plate)	Each well (12-well plate)
All-in-one pAAV–CasRx–pre-sgRNA plasmid (ng)	1,600	800
Lipofectamine 2000 (μl) [DNA (μg)/Lipofectamine 2000 (μl) = 1:3]	4.8	2.4
Opti-MEM (μl)	300	100

(ii) The transfection steps are as follows:

Step 1. Mix DNA in 150 μl (for the 6-well plate) or 50 μl (for the 12-well plate) OptiMEM.
Step 2. Mix Lipofectamine 2000 in 150 μl (for the 6-well plate) or 50 μl (for the 12-well plate) OptiMEM.
Step 3. Mix the DNA/OptiMEM mixture with the Lipofectamine 2000/OptiMEM mixture.
Step 4. Incubate at room temperature for 10 min.
Step 5. Remove the culture media from the plate wells and add 1.7 ml (for the 6-well plate) or 0.9 ml (for the 12-well plate) fresh culture media to each well.
Step 6. Add the DNA/Lipofectamine 2000 mixture to each well dropwise.
Step 7. Incubate at 37°C with 5% CO_2_.
Step 8. Remove the culture media from the plate wells and add 2 ml (for the 6-well plate) or 1 ml (for the 12-well plate) fresh culture media to each well the next day.
Step 9. Incubate at 37°C with 5% CO_2_ for another 48 h.

#### Transfection of Müller Cells

(i) Cells were seeded in multiwell cell culture plates a day before transfection, for the cell density to be at 50–70% during transfection. The ViaFect reagent/DNA mixture is prepared as follows:

**Table:** 

	Each well (six-well plate)	Each well (12-well plate)
All-in-one pAAV–CasRx–pre-sgRNA plasmid (ng)	1,600	800
ViaFect (μl) [DNA (μg)/ViaFect (μl) = 1:6]	9.6	4.8
OptiMEM (μl)	300	100

(ii) The transfection steps are as follows:

Step 1. Mix DNA in 150 μl (for the 6-well plate) or 50 μl (for the 12-well plate) OptiMEM.
Step 2. Mix ViaFect in 150 μl (for the 6-well plate) or 50 μl (for the 12-well plate) OptiMEM.
Step 3. Mix the DNA/OptiMEM mixture with the ViaFect/OptiMEM mixture.
Step 4. Incubate at room temperature for 10 min.
Step 5. Remove the culture media from the plate wells and add 1.7 ml (for the 6-well plate) or 0.9 ml (for the 12-well plate) fresh culture media to each well.
Step 6. Add the DNA/ViaFect mixture to each well dropwise.
Step 7. Incubate at 37°C with 5% CO_2_ for 48 h.

(iii) After transfection, place the samples into GENbag for 24 h.

(iv) For RNA isolation, HEK293FT or Müller cells were harvested using 300 μl RNA lysis buffer and total RNA was isolated using Quick-RNA^®^ Miniprep kit (Zymo Research) and eluted in 10 mM Tris–HCl, pH 8 (or the elution buffer from the kit) (Supplementary Method—Quick-RNA^®^ Miniprep kit). RNA was quantified using Nanodrop^TM^.

It should be noted that (1) cells must not reach full confluency prior to transfection and (2) as RNA is unstable, the process must be performed as quickly as possible.

### Reverse Transcription and Multiplex qPCR

(i) After RNA extraction, 200 ng of RNA/sample was used for reverse transcription. Reverse transcription was performed using High-Capacity cDNA Reverse Transcription kit (Applied Biosystems^TM^). The reverse transcription master mix was prepared as shown below:

**Table:** 

Component	Amount/reaction (μl)
10 × RT buffer	1
dNTP	0.4
10 × RT random primer	1
Reverse transcriptase	0.5
RNase-free water	2.1
Final volume	5

(ii) Five microliters of the master mix was added to 5 μl of RNA (200 ng) to make a 10-μl reverse transcription reaction working solution. Reverse transcription was done with the PCR thermal cycling conditions shown below.

**Table:** 

	Temperature (°C)	Time (min)
Step 1	25	10
Step 2	37	120
Step 3	85	5
Step 4	4	∞

(iii) After reverse transcription, qPCR was done with TaqMan^TM^ Fast Advanced Master Mix (Applied Biosystems^TM^). cDNA was diluted with nuclease-free water in a ratio of 1:9. The qPCR master mix was prepared as shown below.

**Table:** 

Component	Amount/reaction (μl)
TaqMan Fast Advanced Master Mix	5
TaqMan primer (GAPDH, dilute with DNase-free water in a ratio of 1:3)	0.5
TaqMan primer (human VEGFA)	0.5
Nuclease-free water	2
Final volume	8

(iv) Then, 8 μl of the master mix was added to 2 μl of diluted cDNA to make 10 μl of qPCR working solution. qPCR was then performed with a quantitative chain reaction instrument with the thermal cycling conditions shown below.

**Table:** 

	Temperature (°C)	Time (s)
**Hold stage**		
Step 1	95	20
**PCR stage (40 cycles)**		
Step 1	95	1
Step 2	60	20

For troubleshooting: Low RNA knockdown rates might be due to insufficient sgRNA to target mRNA. Consider designing new sgRNAs.

## Results

### Cloning-Free Method for CasRx-Mediated RNA Knockdown

Transgene expression studies typically require cloning of the transgene in plasmids with bacteria, a process that involves restriction enzyme digestion and ligation, bacterial transformation, screening of clones, plasmid DNA purification, and verification of clones (Fang et al., 2019). This tedious and expensive procedure can take up to a week to complete. Cloning-free CRISPR was first described using short fragments of sgRNA (gBlocks^TM^) that consisted of a promoter, sgRNA sequence, and sgRNA hairpin sequence. Knockdown efficiencies comparable to the standard plasmid DNA form of sgRNA were reported (Arbab et al., 2015). Similarly, we demonstrate CasRx-mediated knockdown of VEGFA by delivering sgRNAs in commercially synthesized gBlocks^TM^, which circumvents the need for cloning sgRNAs into plasmids prior to transfection for gene knockdown studies.

**FIGURE 2:**
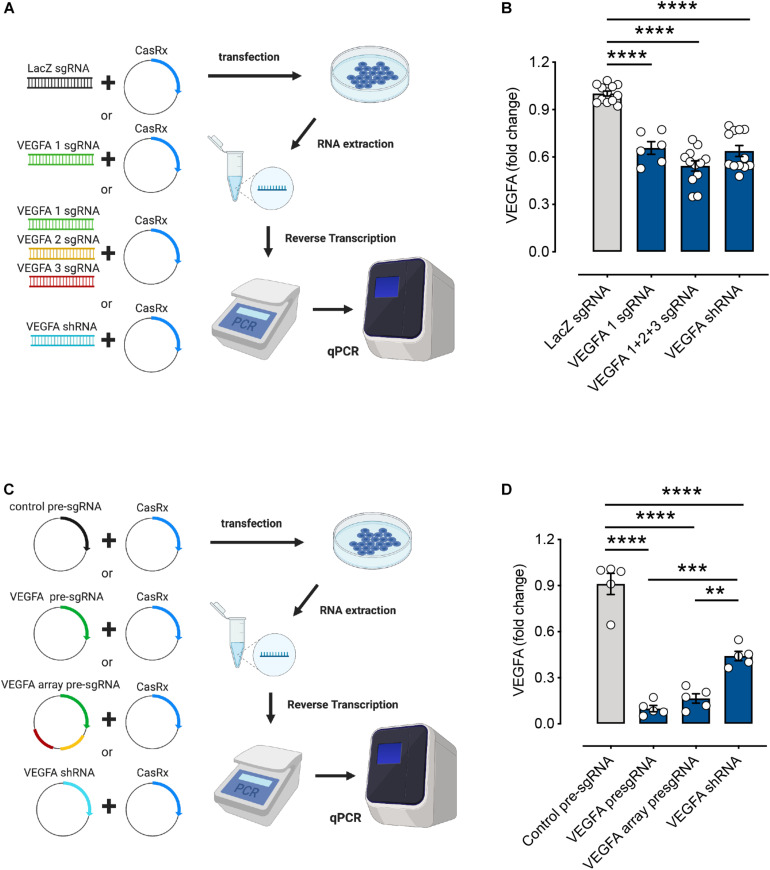
RNA editing efficiencies of the CasRx system using different forms of single guide RNAs (sgRNA). **(A)** HEK293FT cells were transfected with the CasRx plasmid and short fragments of LacZ sgRNA (control), single vascular endothelial growth factor A (VEGFA) sgRNA, mixture of VEGFA sgRNAs (array), or VEGFA short hairpin (shRNA) for 72 h. **(B)** The *VEGFA* mRNA levels were determined by quantitative PCR (qPCR) as described in section “METHODS” (*n* = 6–12). **(C)** HEK293FT cells were transfected with the CasRx plasmid and non-targeting (control) pre-sgRNA, VEGFA pre-sgRNA, VEGFA array pre-sgRNA, and VEGFA shRNA for 72 h. **(D)** The *VEGFA* mRNA level was determined by qPCR as described in section “METHODS” (*n* = 5). Data are expressed as the mean ± SEM. Statistical analysis was performed with GraphPad Prism 7 (GraphPad, San Diego, CA, United States) and undertaken with one-way ANOVA and Tukey’s multiple comparison test. **P < 0.01, ***P < 0.001, ****P < 0.0001. Created with BioRender.com.

To compare the RNA knockdown efficiencies between using a single sgRNA and multiple sgRNAs with the CRISPR/CasRx system, we designed gBlocks^TM^ encoding a single sgRNA and the mixture of multiple sgRNAs to target *VEGFA* mRNA (Figure 2A). We also used sgRNA targeting LacZ mRNA as a negative control and VEGFA-targeting shRNA as a positive control. CasRx significantly knocked down *VEGFA* expression using either gBlocks^TM^ of single sgRNA (34 ± 4% reduction) or multiple sgRNAs (46 ± 3% reduction). Using multiple sgRNAs resulted in a slightly higher RNA knockdown. This was comparable to RNA knockdown by shRNA at 36 ± 4% (Figure 2B).

### Vector Form of Pre-sgRNA Gives Higher RNA Knockdown Rates Compared to gBlocks^TM^

Pre-sgRNAs were described as 30-nt sgRNAs flanked upstream and downstream by full-length 36-nt DRs. This is said to mimic unprocessed guide RNAs. Processed gRNAs were predicted to be 22-nt spacers with a single 30-nt DR upstream (Konermann et al., 2018). To investigate CasRx-mediated knockdown of *VEGFA* mRNA using pre-sgRNA and pre-sgRNA array (encoding an array of three pre-sgRNAs), we designed and cloned VEGFA-targeting pre-sgRNAs into plasmid to compare the RNA knockdown efficiencies with varied formats of sgRNAs (Figure 2C). Non-targeting pre-sgRNA was used as a negative control and the plasmid form of VEGFA shRNA used as a positive control. *VEGFA* mRNA knockdown using single pre-sgRNA (90 ± 2%) and pre-sgRNA array (83 ± 3%) stood at ∼90% compared to shRNA-mediated knockdown at 56 ± 3% (Figure 2D). RNA interference-based methods including siRNA and shRNA have been used widely for gene manipulation (Wilson and Doudna, 2013). Studies have shown that the CRISPR/Cas13 system possesses greater accuracy and efficiency than the RNA interference (RNAi) system and is safer with no detectable off-targets (Abudayyeh et al., 2017; Konermann et al., 2018). Here, our results showed that CasRx-mediated knockdown of *VEGFA* mRNA using pre-sgRNA and pre-sgRNA array is more useful and efficient than the existing RNAi methods. Moreover, the RNA knockdown rates were higher when using pre-sgRNA plasmids compared to gBlocks^TM^ (∼90% *vs*. ∼40% knockdown). In addition, without CasRx, no effect was observed in the *VEGFA* mRNA levels, indicating CasRx to be essential for RNA editing and efficient knockdown of target gene expression (Supplementary Figure 12).

### All-in-One AAV Vector Containing Multiple Pre-sgRNAs With CasRx Shows Efficient RNA Knockdown

Adeno-associated virus-based delivery is preferred among the virus-based delivery methods for basic research and therapeutic applications. It elicits minor immune responses, and with a wide range of AAV serotypes, AAV-based delivery can be made both safe and efficient (Kotterman and Schaffer, 2014; Nathwani et al., 2014; Nelson and Gersbach, 2016). However, the packaging capacity of AAV (∼4.7 Kb) remains limited. CasRx, with a size of 930 aa, can help overcome this challenge. CasRx can also process its own CRISPR array, thereby making the delivery of multiple sgRNAs within a single AAV possible. Using an array to encode four sgRNAs with CasRx, over 90% knockdown of *B4GALNT1* and *ANXA4* mRNA was achieved (Konermann et al., 2018).

**FIGURE 3:**
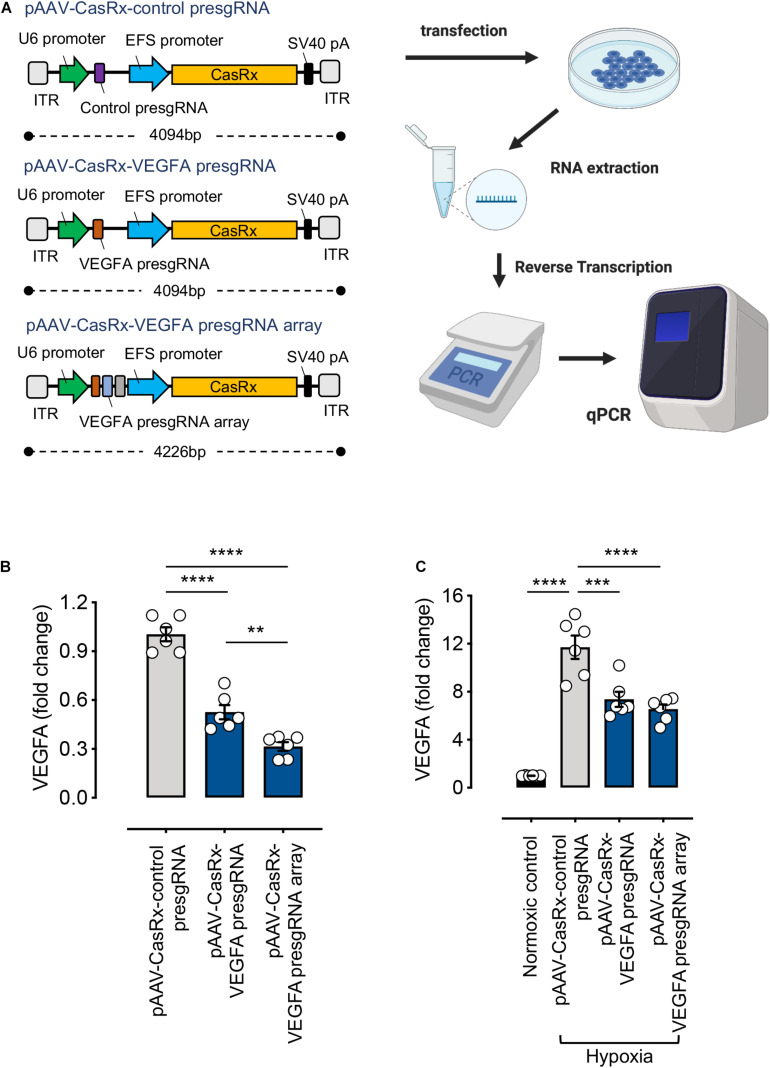
RNA-editing efficiency of the CRISPR/CasRx system using the all-in-one pAAV construct. **(A)** Illustration of the all-in-one pAAV constructs and the experimental procedure. Created with BioRender.com. **(B)** HEK293FT cells were transfected with pAAV–CasRx–control pre-sgRNA, pAAV–CasRx–VEGFA pre-sgRNA, and pAAV–CasRx–VEGFA pre-sgRNA array for 72 h. The *VEGFA* mRNA level was determined by quantitative PCR (qPCR) as described in section “METHODS” (*n* = 6). **(C)** Human Müller cells (MIO-M1) were transfected with pAAV–CasRx–control pre-sgRNA, pAAV–CasRx–VEGFA pre-sgRNA, and pAAV–CasRx–VEGFA pre-sgRNA array for 48 h and under hypoxia condition for another 24 h. The *VEGFA* mRNA level was determined by qPCR as described in section “METHODS” (*n* = 6). Data are expressed as the mean ± SEM. Statistical analysis was performed with GraphPad Prism 7 (GraphPad, San Diego, CA, United States) and undertaken with one-way ANOVA and Tukey’s multiple comparison test. **P < 0.01, ***P < 0.001, ****P < 0.0001.

Here, we examined the RNA knockdown efficiencies of all-in-one CasRx constructs consisting of a single or an array of three pre-sgRNA sequences (pAAV–CasRx–VEGFA pre-sgRNA or pAAV–CasRx–VEGFA pre-sgRNA array) (Figure 3A). An EFS promoter is used to drive the expression of CasRx. Non-targeting pre-sgRNA was used as a negative control (pAAV–CasRx–control pre-sgRNA). Successful knockdown of VEGFA expression was achieved using both single pre-sgRNA (47 ± 4% reduction) and pre-sgRNA array (68 ± 3% reduction) plasmids. Using multiple pre-sgRNAs (array), we achieved higher RNA knockdown efficiencies compared to using a single pre-sgRNA (Figure 3B), corresponding to the study by Konermann et al. (2018). While the RNA knockdown efficiencies achieved stood at 68%, this could be attributed to the use of a weak promoter. A stronger promoter (e.g., CMV) is expected to produce higher knockdown efficiencies while retaining delivery through a single AAV vector.

In addition, to extend the application of this system, we transfected the all-in-one pAAV plasmids into Müller cells. Müller cells are the principal glial cells and supporting cells of the retina, essential for maintaining retinal integrity and homeostasis (Goldman, 2014). *VEGF* is predominantly expressed in certain retinal cell types, including Müller cells (Wang et al., 2010). Especially, large amounts of VEGF are expressed from Müller cells during hypoxia, directly linking Müller-derived VEGF to retinal neovascularization (Aiello et al., 1995). Therefore, we examined the RNA knockdown efficiencies of the all-in-one CasRx constructs under hypoxic condition in Müller cells. Our results showed the *VEGFA* expression to be much higher under hypoxic condition compared to the normoxic control. Our RNA-targeting construct, when delivered through the all-in-one pAAV plasmids, can successfully suppress the increased *VEGFA* expression induced by hypoxia (Figure 3C). Demonstration of the *VEGFA* control in Müller cells suggests the clinical applicability of our RNA-targeting CRISPR/CasRx system.

## Discussion

Here, we describe a detailed step-by-step protocol for designing and validating the CRISPR/CasRx-mediated RNA editing *in vitro*. Our results show that delivering pre-sgRNAs as plasmids with CasRx produced superior RNA knockdown efficiencies than using commercially synthesized short fragments (gBlocks^TM^) of sgRNA. While using plasmids of pre-sgRNA is suitable for most applications, gBlocks^TM^ is still a convenient, low-cost yet powerful method for the validation of RNA knockdown efficiency. To our knowledge, a complete step-by-step protocol for performing RNA knockdown using CRISPR/CasRx is not available from current literature. We also show for the first time that an array of up to three pre-sgRNAs can be incorporated into a single AAV vector with CasRx for successful knockdown of target mRNA while maintaining significant efficiency. CasRx has been previously shown to outperform siRNA-mediated RNAi knockdown (Konermann et al., 2018). shRNA, on the other hand, provides a superior alternative in terms of potency, specificity, and long-term effect compared to siRNA (Rao et al., 2009); however, it requires complex design methods (Jinek et al., 2010). We have demonstrated superior knockdown efficiency to shRNA knockdown using our CRISPR/CasRx system as well, combining simple design with potent RNA knockdown. Delivery through AAV plasmids also ensures prolonged expression and, unlike shRNA, does not integrate into the genome (Duan et al., 1998). Put together, our results describe a safe, compact yet powerful RNA knockdown system compared to existing RNA-targeting methods.

With RNA-targeting systems emerging as potential therapeutic models for the treatment of ocular disease (Zhou et al., 2020; Kantor et al., 2021; Nguyen et al., 2021), a stepwise manual would be instrumental for subsequent *in vivo* studies. In addition, Cas13 systems have been shown potent against RNA viruses, including SARS-COV-2. Recently, CRISPR/CasRx was also shown effective in inducing the apoptosis of cancer cell lines and inhibiting tumor cell growth, establishing itself as a versatile therapeutic platform with antiviral and antitumor potential (Li et al., 2021). Therefore, the methods described herein could facilitate the development of future RNA-targeted therapeutics for multiple diseases (Freije et al., 2019; Zhang, 2020). In conclusion, this paper showcases the breadth of capacities possessed by the CasRx RNA-editing platform and establishes the single-AAV delivery method with CasRx as a viable option for therapeutic purposes.

## Data Availability Statement

The original contributions presented in the study are included in the article/Supplementary Material, further inquiries can be directed to the corresponding author/s.

## Author Contributions

Y-FC and G-SL conceptualized the study, helped with the methodology and project administration, did formal analysis, curated the data, and wrote the original draft. Y-FC, SL, and F-LL contributed to the investigation. P-YW, SK, and F-LL contributed to the writing (review and editing). P-YW and G-SL contributed to funding acquisition and supervised the project. All authors contributed to the article and approved the submitted version.

## Conflict of Interest

The authors declare that the research was conducted in the absence of any commercial or financial relationships that could be construed as a potential conflict of interest.

## Footnotes

1 https://www.ncbi.nlm.nih.gov

2 http://rna.tbi.univie.ac.at//cgi-bin/RNAWebSuite/RNAfold.cgi

3 https://www.benchling.com

## Supplementary Material

Supplementary Table 1 Nucleotide sequences of PCR primers, CasRx plasmid, sgRNA in short fragment form (gBlocks^TM^) or plasmid form, and all-in-one construct form (CasRx and pre-sgRNA in single pAAV). sgRNA sequences are shown in bold.

Supplementary Table 2 List of plasmids and restriction enzymes used for plasmid validation with buffer used for respective restriction enzymes.
