# Supplementary material for: Methods for in vitro CRISPR/CasRx-Mediated RNA Editing
Source: Front Cell Dev Biol. 2021 Jun 11;9:667879. doi: 10.3389/fcell.2021.667879 (PMC8226256; doi:10.3389/fcell.2021.667879)
Supplement: Supplementary Table 1 — Nucleotide sequences of PCR primers, CasRx plasmid, sgRNA in short fragment form (gBlocksTM) or plasmid form, and all-in-one construct form (CasRx and pre-sgRNA in single pAAV). sgRNA sequences are shown in bold. [file Table_1.docx]

**SUPPLEMENTARY TABLES**

**TABLES S1-S2**

**Table S1** Nucleotide sequences of PCR primers, CasRx plasmid, sgRNA in short fragment form (gBlocks™) or plasmid form, and all-in-one construct form (CasRx and pre-sgRNA in single pAAV). sgRNA sequences are shown in bold.

| **Descriptions** | **Sequences** |
| --- | --- |
| sgRNA cassette Forward primer (IDT™ oligo) | TGAGTATTACGGCATGTGAGGGC |
| sgRNA cassette Reverse primer (IDT™ oligo) | TCAATGTATCTTATCATGTCTGCTCGA |
| Sequencing primer (LKO.1) | gactatcatatgcttaccgt |
| LacZ sgRNA- gBlocks™ | TGAGTATTACGGCATGTGAGGGCCTATTTCCCATGATTCCTTCATATTTGCATATACGATACAAGGCTGTTAGAGAGATAATTGGAATTAATTTGACTGTAAACACAAAGATATTAGTACAAAATACGTGACGTAGAAAGTAATAATTTCTTGGGTAGTTTGCAGTTTTAAAATTATGTTTTAAAATGGACTATCATATGCTTACCGTAACTTGAAAGTATTTCGATTTCTTGGCTTTATATATCTTGTGGAAAGGACGAAACACCGAACCCCTACCAACTGGTCGGGGTTTGAAAC**GTTGCGAATACGCCCACGCGAT**TTTTTTTGAATTCTGATGCGGTATTTTCTCCTTACGCATCTGTGCGGTATTTCACACCGCATACGTCAAAGCAACCATAGTTCGAGCAGACATGATAAGATACATTGA |
| VEGFA sgRNA1- gBlocks™ | GCGGCCGCTGAGTATTACGGCATGTGAGGGCCTATTTCCCATGATTCCTTCATATTTGCATATACGATACAAGGCTGTTAGAGAGATAATTGGAATTAATTTGACTGTAAACACAAAGATATTAGTACAAAATACGTGACGTAGAAAGTAATAATTTCTTGGGTAGTTTGCAGTTTTAAAATTATGTTTTAAAATGGACTATCATATGCTTACCGTAACTTGAAAGTATTTCGATTTCTTGGCTTTATATATCTTGTGGAAAGGACGAAACACCGAACCCCTACCAACTGGTCGGGGTTTGAAAC**TATGTGCTGGCCTTGGTGAGGT**TTTTTTTGAATTCTGATGCGGTATTTTCTCCTTACGCATCTGTGCGGTATTTCACACCGCATACGTCAAAGCAACCATAGTTCGAGCAGACATGATAAGATACATTGACTCGAG |
| VEGFA sgRNA2- gBlocks™ | TGAGTATTACGGCATGTGAGGGCCTATTTCCCATGATTCCTTCATATTTGCATATACGATACAAGGCTGTTAGAGAGATAATTGGAATTAATTTGACTGTAAACACAAAGATATTAGTACAAAATACGTGACGTAGAAAGTAATAATTTCTTGGGTAGTTTGCAGTTTTAAAATTATGTTTTAAAATGGACTATCATATGCTTACCGTAACTTGAAAGTATTTCGATTTCTTGGCTTTATATATCTTGTGGAAAGGACGAAACACCGAACCCCTACCAACTGGTCGGGGTTTGAAAC**CACTTCGTGATGATTCTGCCCT**TTTTTTTGAATTCTGATGCGGTATTTTCTCCTTACGCATCTGTGCGGTATTTCACACCGCATACGTCAAAGCAACCATAGTTCGAGCAGACATGATAAGATACATTGA |
| VEGFA sgRNA3- gBlocks™ | TGAGTATTACGGCATGTGAGGGCCTATTTCCCATGATTCCTTCATATTTGCATATACGATACAAGGCTGTTAGAGAGATAATTGGAATTAATTTGACTGTAAACACAAAGATATTAGTACAAAATACGTGACGTAGAAAGTAATAATTTCTTGGGTAGTTTGCAGTTTTAAAATTATGTTTTAAAATGGACTATCATATGCTTACCGTAACTTGAAAGTATTTCGATTTCTTGGCTTTATATATCTTGTGGAAAGGACGAAACACCGAACCCCTACCAACTGGTCGGGGTTTGAAAC**TCTGCATTCACATTTGTTGTGC**TTTTTTTGAATTCTGATGCGGTATTTTCTCCTTACGCATCTGTGCGGTATTTCACACCGCATACGTCAAAGCAACCATAGTTCGAGCAGACATGATAAGATACATTGA |
| VEGFA shRNA- gBlocks™ | GCGGCCGCTGAGTATTACGGCATGTGAGGGCCTATTTCCCATGATTCCTTCATATTTGCATATACGATACAAGGCTGTTAGAGAGATAATTGGAATTAATTTGACTGTAAACACAAAGATATTAGTACAAAATACGTGACGTAGAAAGTAATAATTTCTTGGGTAGTTTGCAGTTTTAAAATTATGTTTTAAAATGGACTATCATATGCTTACCGTAACTTGAAAGTATTTCGATTTCTTGGCTTTATATATCTTGTGGAAAGGACGAAAGCGGTGCTGGCCTTGGTGAGGTTCAAGAGACCTCACCAAGGCCAGCACTTTTTTTTGATGCGGTATTTTCTCCTTACGCATCTGTGCGGTATTTCACACCGCATACGTCAAAGCAACCATAGTTCGAGCAGACATGATAAGATACATTGACTCGAG |
| Control (non-targeting) presgRNA cassette for Gibson assembly- gBlocks™ | ggccttttgctggccttttgctcagctaggagggcctatttcccatgattccttcatatttgcatatacgatacaaggctgttagagagataattggaattaatttgactgtaaacacaaagatattagtacaaaatacgtgacgtagaaagtaataatttcttgggtagtttgcagttttaaaattatgttttaaaatggactatcatatgcttaccgtaacttgaaagtatttcgatttcttggctttatatatcttgtggaaaggacgaaacaccgcaagtaaacccctaccaactggtcggggtttgaaac**TCACCAGAAGCGTACCATACTCACGAACAG**caagtaaacccctaccaactggtcggggtttgaaactttttttgaattctgatgcggtattttctccttacgc |
| VEGFA presgRNA cassette for Gibson assembly- gBlocks™ | ggccttttgctggccttttgctcagctaggagggcctatttcccatgattccttcatatttgcatatacgatacaaggctgttagagagataattggaattaatttgactgtaaacacaaagatattagtacaaaatacgtgacgtagaaagtaataatttcttgggtagtttgcagttttaaaattatgttttaaaatggactatcatatgcttaccgtaacttgaaagtatttcgatttcttggctttatatatcttgtggaaaggacgaaacaccgcaagtaaacccctaccaactggtcggggtttgaaac**tatgtgctggccttggtgaggtttgatccg**caagtaaacccctaccaactggtcggggtttgaaactttttttgaattctgatgcggtattttctccttacgc |
| VEGFA presgRNA array cassette for Gibson assembly- gBlocks™ | ggccttttgctggccttttgctcagctaggagggcctatttcccatgattccttcatatttgcatatacgatacaaggctgttagagagataattggaattaatttgactgtaaacacaaagatattagtacaaaatacgtgacgtagaaagtaataatttcttgggtagtttgcagttttaaaattatgttttaaaatggactatcatatgcttaccgtaacttgaaagtatttcgatttcttggctttatatatcttgtggaaaggacgaaacaccgcaagtaaacccctaccaactggtcggggtttgaaac**tatgtgctggccttggtgaggtttgatccg**caagtaaacccctaccaactggtcggggtttgaaac**cacttcgtgatgattctgccctcctccttc**caagtaaacccctaccaactggtcggggtttgaaact**ctgcattcacatttgttgtgctgtaggaa**caagtaaacccctaccaactggtcggggtttgaaactttttttgaattctgatgcggtattttctccttacgc |
| VEGFA shRNA cassette for Gibson assembly-gBlocks™ | ggccttttgctggccttttgctcagctaggagggcctatttcccatgattccttcatatttgcatatacgatacaaggctgttagagagataattggaattaatttgactgtaaacacaaagatattagtacaaaatacgtgacgtagaaagtaataatttcttgggtagtttgcagttttaaaattatgttttaaaatggactatcatatgcttaccgtaacttgaaagtatttcgatttcttggctttatatatcttgtggaaaggacgaaacaccg**ACCTCACCAAGGCCAGCACTCAAGAGGTGCTGGCCTTGGTGAGGT**tttttttgaattctgatgcggtattttctccttacgc |
| Control (non-targeting) presgRNA (pM115)- Plasmid DNA | GCTAGCGAGGGCCTATTTCCCATGATTCCTTCATATTTGCATATACGATACAAGGCTGTTAGAGAGATAATTGGAATTAATTTGACTGTAAACACAAAGATATTAGTACAAAATACGTGACGTAGAAAGTAATAATTTCTTGGGTAGTTTGCAGTTTTAAAATTATGTTTTAAAATGGACTATCATATGCTTACCGTAACTTGAAAGTATTTCGATTTCTTGGCTTTATATATCTTGTGGAAAGGACGAAACACCGCAAGTAAACCCCTACCAACTGGTCGGGGTTTGAAAC**TCACCAGAAGCGTACCATACTCACGAACAG**CAAGTAAACCCCTACCAACTGGTCGGGGTTTGAAACTTTTTTTGAATTCTGATGCGGTATTTTCTCCTTACGCATCTGTGCGGTATTTCACACCGCATACGTCAAAGCAACCATAGTACGCGCCCTGTAGCGGCGCATTAAGCGCGGCGGGTGTGGTGGTTACGCGCAGCGTGACCGCTACACTTGCCAGCGCCTTAGCGCCCGCTCCTTTCGCTTTCTTCCCTTCCTTTCTCGCCACGTTCGCCGGCTTTCCCCGTCAAGCTCTAAATCGGGGGCTCCCTTTAGGGTTCCGATTTAGTGCTTTACGGCACCTCGACCCCAAAAAACTTGATTTGGGTGATGGTTCACGTAGTGGGCCATCGCCCTGATAGACGGTTTTTCGCCCTTTGACGTTGGAGTCCACGTTCTTTAATAGTGGACTCTTGTTCCAAACTGGAACAACACTCAACTCTATCTCGGGCTATTCTTTTGATTTATAAGGGATTTTGCCGATTTCGGTCTATTGGTTAAAAAATGAGCTGATTTAACAAAAATTTAACGCGAATTTTAACAAAATATTAACGTTTACAATTTTATGGTGCACTCTCAGTACAATCTGCTCTGATGCCGCATAGTTAAGCCAGCCCCGACACCCGCCAACACCCGCTGACGCGCCCTGACGGGCTTGTCTGCTCCCGGCATCCGCTTACAGACAAGCTGTGACCGTCTCCGGGAGCTGCATGTGTCAGAGGTTTTCACCGTCATCACCGAAACGCGCGAGACGAAAGGGCCTCGTGATACGCCTATTTTTATAGGTTAATGTCATGATAATAATGGTTTCTTAGACGTCAGGTGGCACTTTTCGGGGAAATGTGCGCGGAACCCCTATTTGTTTATTTTTCTAAATACATTCAAATATGTATCCGCTCATGAGACAATAACCCTGATAAATGCTTCAATAATATTGAAAAAGGAAGAGTATGAGTATTCAACATTTCCGTGTCGCCCTTATTCCCTTTTTTGCGGCATTTTGCCTTCCTGTTTTTGCTCACCCAGAAACGCTGGTGAAAGTAAAAGATGCTGAAGATCAGTTGGGTGCACGAGTGGGTTACATCGAACTGGATCTCAACAGCGGTAAGATCCTTGAGAGTTTTCGCCCCGAAGAACGTTTTCCAATGATGAGCACTTTTAAAGTTCTGCTATGTGGCGCGGTATTATCCCGTATTGACGCCGGGCAAGAGCAACTCGGTCGCCGCATACACTATTCTCAGAATGACTTGGTTGAGTACTCACCAGTCACAGAAAAGCATCTTACGGATGGCATGACAGTAAGAGAATTATGCAGTGCTGCCATAACCATGAGTGATAACACTGCGGCCAACTTACTTCTGACAACGATCGGAGGACCGAAGGAGCTAACCGCTTTTTTGCACAACATGGGGGATCATGTAACTCGCCTTGATCGTTGGGAACCGGAGCTGAATGAAGCCATACCAAACGACGAGCGTGACACCACGATGCCTGTAGCAATGGCAACAACGTTGCGCAAACTATTAACTGGCGAACTACTTACTCTAGCTTCCCGGCAACAATTAATAGACTGGATGGAGGCGGATAAAGTTGCAGGACCACTTCTGCGCTCGGCCCTTCCGGCTGGCTGGTTTATTGCTGATAAATCTGGAGCCGGTGAGCGTGGAAGCCGCGGTATCATTGCAGCACTGGGGCCAGATGGTAAGCCCTCCCGTATCGTAGTTATCTACACGACGGGGAGTCAGGCAACTATGGATGAACGAAATAGACAGATCGCTGAGATAGGTGCCTCACTGATTAAGCATTGGTAACTGTCAGACCAAGTTTACTCATATATACTTTAGATTGATTTAAAACTTCATTTTTAATTTAAAAGGATCTAGGTGAAGATCCTTTTTGATAATCTCATGACCAAAATCCCTTAACGTGAGTTTTCGTTCCACTGAGCGTCAGACCCCGTAGAAAAGATCAAAGGATCTTCTTGAGATCCTTTTTTTCTGCGCGTAATCTGCTGCTTGCAAACAAAAAAACCACCGCTACCAGCGGTGGTTTGTTTGCCGGATCAAGAGCTACCAACTCTTTTTCCGAAGGTAACTGGCTTCAGCAGAGCGCAGATACCAAATACTGTTCTTCTAGTGTAGCCGTAGTTAGGCCACCACTTCAAGAACTCTGTAGCACCGCCTACATACCTCGCTCTGCTAATCCTGTTACCAGTGGCTGCTGCCAGTGGCGATAAGTCGTGTCTTACCGGGTTGGACTCAAGACGATAGTTACCGGATAAGGCGCAGCGGTCGGGCTGAACGGGGGGTTCGTGCACACAGCCCAGCTTGGAGCGAACGACCTACACCGAACTGAGATACCTACAGCGTGAGCTATGAGAAAGCGCCACGCTTCCCGAAGGGAGAAAGGCGGACAGGTATCCGGTAAGCGGCAGGGTCGGAACAGGAGAGCGCACGAGGGAGCTTCCAGGGGGAAACGCCTGGTATCTTTATAGTCCTGTCGGGTTTCGCCACCTCTGACTTGAGCGTCGATTTTTGTGATGCTCGTCAGGGGGGCGGAGCCTATGGAAAAACGCCAGCAACGCGGCCTTTTTACGGTTCCTGGCCTTTTGCTGGCCTTTTGCTCA |
| VEGFA presgRNA (pM116)- Plasmid DNA | GCTAGCGAGGGCCTATTTCCCATGATTCCTTCATATTTGCATATACGATACAAGGCTGTTAGAGAGATAATTGGAATTAATTTGACTGTAAACACAAAGATATTAGTACAAAATACGTGACGTAGAAAGTAATAATTTCTTGGGTAGTTTGCAGTTTTAAAATTATGTTTTAAAATGGACTATCATATGCTTACCGTAACTTGAAAGTATTTCGATTTCTTGGCTTTATATATCTTGTGGAAAGGACGAAACACCGCAAGTAAACCCCTACCAACTGGTCGGGGTTTGAAAC**TATGTGCTGGCCTTGGTGAGGTTTGATCCG**CAAGTAAACCCCTACCAACTGGTCGGGGTTTGAAACTTTTTTTGAATTCTGATGCGGTATTTTCTCCTTACGCATCTGTGCGGTATTTCACACCGCATACGTCAAAGCAACCATAGTACGCGCCCTGTAGCGGCGCATTAAGCGCGGCGGGTGTGGTGGTTACGCGCAGCGTGACCGCTACACTTGCCAGCGCCTTAGCGCCCGCTCCTTTCGCTTTCTTCCCTTCCTTTCTCGCCACGTTCGCCGGCTTTCCCCGTCAAGCTCTAAATCGGGGGCTCCCTTTAGGGTTCCGATTTAGTGCTTTACGGCACCTCGACCCCAAAAAACTTGATTTGGGTGATGGTTCACGTAGTGGGCCATCGCCCTGATAGACGGTTTTTCGCCCTTTGACGTTGGAGTCCACGTTCTTTAATAGTGGACTCTTGTTCCAAACTGGAACAACACTCAACTCTATCTCGGGCTATTCTTTTGATTTATAAGGGATTTTGCCGATTTCGGTCTATTGGTTAAAAAATGAGCTGATTTAACAAAAATTTAACGCGAATTTTAACAAAATATTAACGTTTACAATTTTATGGTGCACTCTCAGTACAATCTGCTCTGATGCCGCATAGTTAAGCCAGCCCCGACACCCGCCAACACCCGCTGACGCGCCCTGACGGGCTTGTCTGCTCCCGGCATCCGCTTACAGACAAGCTGTGACCGTCTCCGGGAGCTGCATGTGTCAGAGGTTTTCACCGTCATCACCGAAACGCGCGAGACGAAAGGGCCTCGTGATACGCCTATTTTTATAGGTTAATGTCATGATAATAATGGTTTCTTAGACGTCAGGTGGCACTTTTCGGGGAAATGTGCGCGGAACCCCTATTTGTTTATTTTTCTAAATACATTCAAATATGTATCCGCTCATGAGACAATAACCCTGATAAATGCTTCAATAATATTGAAAAAGGAAGAGTATGAGTATTCAACATTTCCGTGTCGCCCTTATTCCCTTTTTTGCGGCATTTTGCCTTCCTGTTTTTGCTCACCCAGAAACGCTGGTGAAAGTAAAAGATGCTGAAGATCAGTTGGGTGCACGAGTGGGTTACATCGAACTGGATCTCAACAGCGGTAAGATCCTTGAGAGTTTTCGCCCCGAAGAACGTTTTCCAATGATGAGCACTTTTAAAGTTCTGCTATGTGGCGCGGTATTATCCCGTATTGACGCCGGGCAAGAGCAACTCGGTCGCCGCATACACTATTCTCAGAATGACTTGGTTGAGTACTCACCAGTCACAGAAAAGCATCTTACGGATGGCATGACAGTAAGAGAATTATGCAGTGCTGCCATAACCATGAGTGATAACACTGCGGCCAACTTACTTCTGACAACGATCGGAGGACCGAAGGAGCTAACCGCTTTTTTGCACAACATGGGGGATCATGTAACTCGCCTTGATCGTTGGGAACCGGAGCTGAATGAAGCCATACCAAACGACGAGCGTGACACCACGATGCCTGTAGCAATGGCAACAACGTTGCGCAAACTATTAACTGGCGAACTACTTACTCTAGCTTCCCGGCAACAATTAATAGACTGGATGGAGGCGGATAAAGTTGCAGGACCACTTCTGCGCTCGGCCCTTCCGGCTGGCTGGTTTATTGCTGATAAATCTGGAGCCGGTGAGCGTGGAAGCCGCGGTATCATTGCAGCACTGGGGCCAGATGGTAAGCCCTCCCGTATCGTAGTTATCTACACGACGGGGAGTCAGGCAACTATGGATGAACGAAATAGACAGATCGCTGAGATAGGTGCCTCACTGATTAAGCATTGGTAACTGTCAGACCAAGTTTACTCATATATACTTTAGATTGATTTAAAACTTCATTTTTAATTTAAAAGGATCTAGGTGAAGATCCTTTTTGATAATCTCATGACCAAAATCCCTTAACGTGAGTTTTCGTTCCACTGAGCGTCAGACCCCGTAGAAAAGATCAAAGGATCTTCTTGAGATCCTTTTTTTCTGCGCGTAATCTGCTGCTTGCAAACAAAAAAACCACCGCTACCAGCGGTGGTTTGTTTGCCGGATCAAGAGCTACCAACTCTTTTTCCGAAGGTAACTGGCTTCAGCAGAGCGCAGATACCAAATACTGTTCTTCTAGTGTAGCCGTAGTTAGGCCACCACTTCAAGAACTCTGTAGCACCGCCTACATACCTCGCTCTGCTAATCCTGTTACCAGTGGCTGCTGCCAGTGGCGATAAGTCGTGTCTTACCGGGTTGGACTCAAGACGATAGTTACCGGATAAGGCGCAGCGGTCGGGCTGAACGGGGGGTTCGTGCACACAGCCCAGCTTGGAGCGAACGACCTACACCGAACTGAGATACCTACAGCGTGAGCTATGAGAAAGCGCCACGCTTCCCGAAGGGAGAAAGGCGGACAGGTATCCGGTAAGCGGCAGGGTCGGAACAGGAGAGCGCACGAGGGAGCTTCCAGGGGGAAACGCCTGGTATCTTTATAGTCCTGTCGGGTTTCGCCACCTCTGACTTGAGCGTCGATTTTTGTGATGCTCGTCAGGGGGGCGGAGCCTATGGAAAAACGCCAGCAACGCGGCCTTTTTACGGTTCCTGGCCTTTTGCTGGCCTTTTGCTCA |
| VEGFA presgRNA array (pM117)- Plasmid DNA | GCTAGCGAGGGCCTATTTCCCATGATTCCTTCATATTTGCATATACGATACAAGGCTGTTAGAGAGATAATTGGAATTAATTTGACTGTAAACACAAAGATATTAGTACAAAATACGTGACGTAGAAAGTAATAATTTCTTGGGTAGTTTGCAGTTTTAAAATTATGTTTTAAAATGGACTATCATATGCTTACCGTAACTTGAAAGTATTTCGATTTCTTGGCTTTATATATCTTGTGGAAAGGACGAAACACCGCAAGTAAACCCCTACCAACTGGTCGGGGTTTGAAAC**TATGTGCTGGCCTTGGTGAGGTTTGATCCG**CAAGTAAACCCCTACCAACTGGTCGGGGTTTGAAAC**CACTTCGTGATGATTCTGCCCTCCTCCTTC**CAAGTAAACCCCTACCAACTGGTCGGGGTTTGAAAC**TCTGCATTCACATTTGTTGTGCTGTAGGAA**CAAGTAAACCCCTACCAACTGGTCGGGGTTTGAAACTTTTTTTGAATTCTGATGCGGTATTTTCTCCTTACGCATCTGTGCGGTATTTCACACCGCATACGTCAAAGCAACCATAGTACGCGCCCTGTAGCGGCGCATTAAGCGCGGCGGGTGTGGTGGTTACGCGCAGCGTGACCGCTACACTTGCCAGCGCCTTAGCGCCCGCTCCTTTCGCTTTCTTCCCTTCCTTTCTCGCCACGTTCGCCGGCTTTCCCCGTCAAGCTCTAAATCGGGGGCTCCCTTTAGGGTTCCGATTTAGTGCTTTACGGCACCTCGACCCCAAAAAACTTGATTTGGGTGATGGTTCACGTAGTGGGCCATCGCCCTGATAGACGGTTTTTCGCCCTTTGACGTTGGAGTCCACGTTCTTTAATAGTGGACTCTTGTTCCAAACTGGAACAACACTCAACTCTATCTCGGGCTATTCTTTTGATTTATAAGGGATTTTGCCGATTTCGGTCTATTGGTTAAAAAATGAGCTGATTTAACAAAAATTTAACGCGAATTTTAACAAAATATTAACGTTTACAATTTTATGGTGCACTCTCAGTACAATCTGCTCTGATGCCGCATAGTTAAGCCAGCCCCGACACCCGCCAACACCCGCTGACGCGCCCTGACGGGCTTGTCTGCTCCCGGCATCCGCTTACAGACAAGCTGTGACCGTCTCCGGGAGCTGCATGTGTCAGAGGTTTTCACCGTCATCACCGAAACGCGCGAGACGAAAGGGCCTCGTGATACGCCTATTTTTATAGGTTAATGTCATGATAATAATGGTTTCTTAGACGTCAGGTGGCACTTTTCGGGGAAATGTGCGCGGAACCCCTATTTGTTTATTTTTCTAAATACATTCAAATATGTATCCGCTCATGAGACAATAACCCTGATAAATGCTTCAATAATATTGAAAAAGGAAGAGTATGAGTATTCAACATTTCCGTGTCGCCCTTATTCCCTTTTTTGCGGCATTTTGCCTTCCTGTTTTTGCTCACCCAGAAACGCTGGTGAAAGTAAAAGATGCTGAAGATCAGTTGGGTGCACGAGTGGGTTACATCGAACTGGATCTCAACAGCGGTAAGATCCTTGAGAGTTTTCGCCCCGAAGAACGTTTTCCAATGATGAGCACTTTTAAAGTTCTGCTATGTGGCGCGGTATTATCCCGTATTGACGCCGGGCAAGAGCAACTCGGTCGCCGCATACACTATTCTCAGAATGACTTGGTTGAGTACTCACCAGTCACAGAAAAGCATCTTACGGATGGCATGACAGTAAGAGAATTATGCAGTGCTGCCATAACCATGAGTGATAACACTGCGGCCAACTTACTTCTGACAACGATCGGAGGACCGAAGGAGCTAACCGCTTTTTTGCACAACATGGGGGATCATGTAACTCGCCTTGATCGTTGGGAACCGGAGCTGAATGAAGCCATACCAAACGACGAGCGTGACACCACGATGCCTGTAGCAATGGCAACAACGTTGCGCAAACTATTAACTGGCGAACTACTTACTCTAGCTTCCCGGCAACAATTAATAGACTGGATGGAGGCGGATAAAGTTGCAGGACCACTTCTGCGCTCGGCCCTTCCGGCTGGCTGGTTTATTGCTGATAAATCTGGAGCCGGTGAGCGTGGAAGCCGCGGTATCATTGCAGCACTGGGGCCAGATGGTAAGCCCTCCCGTATCGTAGTTATCTACACGACGGGGAGTCAGGCAACTATGGATGAACGAAATAGACAGATCGCTGAGATAGGTGCCTCACTGATTAAGCATTGGTAACTGTCAGACCAAGTTTACTCATATATACTTTAGATTGATTTAAAACTTCATTTTTAATTTAAAAGGATCTAGGTGAAGATCCTTTTTGATAATCTCATGACCAAAATCCCTTAACGTGAGTTTTCGTTCCACTGAGCGTCAGACCCCGTAGAAAAGATCAAAGGATCTTCTTGAGATCCTTTTTTTCTGCGCGTAATCTGCTGCTTGCAAACAAAAAAACCACCGCTACCAGCGGTGGTTTGTTTGCCGGATCAAGAGCTACCAACTCTTTTTCCGAAGGTAACTGGCTTCAGCAGAGCGCAGATACCAAATACTGTTCTTCTAGTGTAGCCGTAGTTAGGCCACCACTTCAAGAACTCTGTAGCACCGCCTACATACCTCGCTCTGCTAATCCTGTTACCAGTGGCTGCTGCCAGTGGCGATAAGTCGTGTCTTACCGGGTTGGACTCAAGACGATAGTTACCGGATAAGGCGCAGCGGTCGGGCTGAACGGGGGGTTCGTGCACACAGCCCAGCTTGGAGCGAACGACCTACACCGAACTGAGATACCTACAGCGTGAGCTATGAGAAAGCGCCACGCTTCCCGAAGGGAGAAAGGCGGACAGGTATCCGGTAAGCGGCAGGGTCGGAACAGGAGAGCGCACGAGGGAGCTTCCAGGGGGAAACGCCTGGTATCTTTATAGTCCTGTCGGGTTTCGCCACCTCTGACTTGAGCGTCGATTTTTGTGATGCTCGTCAGGGGGGCGGAGCCTATGGAAAAACGCCAGCAACGCGGCCTTTTTACGGTTCCTGGCCTTTTGCTGGCCTTTTGCTCA |
| VEGFA shRNA- Plasmid DNA | TCGCGCGTTTCGGTGATGACGGTGAAAACCTCTGACACATGCAGCTCCCGGAGACGGTCACAGCTTGTCTGTAAGCGGATGCCGGGAGCAGACAAGCCCGTCAGGGCGCGTCAGCGGGTGTTGGCGGGTGTCGGGGCTGGCTTAACTATGCGGCATCAGAGCAGATTGTACTGAGAGTGCACCAAATGCGGTGTGAAATACCGCACAGATGCGTAAGGAGAAAATACCGCATCAGGCGCCATTCGCCATTCAGGCTGCGCAACTGTTGGGAAGGGCGATCGGTGCGGGCCTCATCGCTATTACGCCAGCTGGCGAAAGGGGGATGTGCTGCAAGGCGATTAAGTTGGGTAACGCCAGGGTTTTCCCAGTCACGACGTTGTAAAACGACGGCCAGTGCAACGCGATGACGATGGATAGCGATTCATCGATGAGCTGACCCGATCGCGGCCGCCGGAGGGTTGCGTTTGAGACGGGCGACAGATGCGGCCGCTGAGTATTACGGCATGTGAGGGCCTATTTCCCATGATTCCTTCATATTTGCATATACGATACAAGGCTGTTAGAGAGATAATTGGAATTAATTTGACTGTAAACACAAAGATATTAGTACAAAATACGTGACGTAGAAAGTAATAATTTCTTGGGTAGTTTGCAGTTTTAAAATTATGTTTTAAAATGGACTATCATATGCTTACCGTAACTTGAAAGTATTTCGATTTCTTGGCTTTATATATCTTGTGGAAAGGACGAAAGCG**ACCTCACCAAGGCCAGCACTCAAGAGGTGCTGGCCTTGGTGAGGT**TTTTTTTTGATGCGGTATTTTCTCCTTACGCATCTGTGCGGTATTTCACACCGCATACGTCAAAGCAACCATAGTTCGAGCAGACATGATAAGATACATTGACTCGAGATCAGTTCTGGACCAGCGAGCTGTGCTGCGACGCGTGGCGTAATCATGGTCATAGCTGTTTCCTGTGTGAAATTGTTATCCGCTCACAATTCCACACAACATACGAGCCGGAAGCATAAAGTGTAAAGCCTGGGGTGCCTAATGAGTGAGCTAACTCACATTAATTGCGTTGCGCTCACTGCCCGCTTTCCAGTCGGGAAACCTGTCGTGCCAGCTGCATTAATGAATCGGCCAACGCGCGGGGAGAGGCGGTTTGCGTATTGGGCGCTCTTTCGCTTCCTCGCTCACTGACTCGCTGCGCTCGGTCGTTCGGCTGCGGCGAGCGGTATCAGCTCACTCAAAGGCGGTAATACGGTTATCCACAGAATCAGGGGATAACGCAGGAAAGAACATGTGAGCAAAAGGCCAGCAAAAGGCCAGGAACCGTAAAAAGGCCGCGTTGCTGGCGTTTTTCCATAGGCTCCGCCCCCCTGACGAGCATCACAAAAATCGACGCTCAAGTCAGAGGTGGCGAAACCCGACAGGACTATAAAGATACCAGGCGTTTCCCCCTGGAAGCTCCCTCGTGCGCTCTCCTGTTCCGACCCTGTCGCTTACCGGATACCTGTCCGCCTTTCTCCCTTCGGGAAGCGTGGCGCTTTCTCATAGCTCACGCTGTAGGTATCTCAGTTCGGTGTAGGTCGTTCGCTCCAAGCTGGGCTGTGTGCACGAACCCCCCGTTCAGCCCGACCGCTGCGCCTTATCCGGTAACTATCGTCTTGAGTCCAACCCGGTAAGACACGACTTATCGCCACTGGCAGCAGCCACTGGTAACAGGATTAGCAGAGCGAGGTATGTAGGCGGTGCTACAGAGTTCTTGAAGTGGTGGCCTAACTACGGCTACACTAGAAGAACAGTATTTGGTATCTGCGCTCTGCTGAAGCCAGTTACCTTCGGAAAAAGAGTTGGTAGCTCTTGATCCGGCAAACAAACCACCGCTGGTAGCGGTGGTTTTTTTGTTTGCAAGCAGCAGATTACGCGCAGAAAAAAAGGATCTCAAGAAGATCCTTTGATCTTTTCTACGGGGTCTGACGCTCAGTGGAACGAAAACTCACGTTAAGGGATTTTGGTCATGAGATTATCAAAAAGGATCTTCACCTAGATCCTTTTAAATTAAAAATGAAGTTTTAAATCAATCTAAAGTATATATGAGTAAACTTGGTCTGACAGTTACCAATGCTTAATCAGTGAGGCACCTATCTCAGCGATCTGTCTATTTCGTTCATCCATAGTTGCCTGACTCCCCGTCGTGTAGATAACTACGATACGGGAGGGCTTACCATCTGGCCCCAGTGCTGCAATGATACCGCGAGACCCACGCTCACCGGCTCCAGATTTATCAGCAATAAACCAGCCAGCCGGAAGGGCCGAGCGCAGAAGTGGTCCTGCAACTTTATCCGCCTCCATCCAGTCTATTAATTGTTGCCGGGAAGCTAGAGTAAGTAGTTCGCCAGTTAATAGTTTGCGCAACGTTGTTGCCATTGCTACAGGCATCGTGGTGTCACGCTCGTCGTTTGGTATGGCTTCATTCAGCTCCGGTTCCCAACGATCAAGGCGAGTTACATGATCCCCCATGTTGTGCAAAAAAGCGGTTAGCTCCTTCGGTCCTCCGATCGTTGTCAGAAGTAAGTTGGCCGCAGTGTTATCACTCATGGTTATGGCAGCACTGCATAATTCTCTTACTGTCATGCCATCCGTAAGATGCTTTTCTGTGACTGGTGAGTACTCAACCAAGTCATTCTGAGAATAGTGTATGCGGCGACCGAGTTGCTCTTGCCCGGCGTCAATACGGGATAATACCGCGCCACATAGCAGAACTTTAAAAGTGCTCATCATTGGAAAACGTTCTTCGGGGCGAAAACTCTCAAGGATCTTACCGCTGTTGAGATCCAGTTCGATGTAACCCACTCGTGCACCCAACTGATCTTCAGCATCTTTTACTTTCACCAGCGTTTCTGGGTGAGCAAAAACAGGAAGGCAAAATGCCGCAAAAAAGGGAATAAGGGCGACACGGAAATGTTGAATACTCATACTCTACCTTTTTCAATATTATTGAAGCATTTATCAGGGTTATTGTCTCATGAGCGGATACATATTTGAATGTATTTAGAAAAATAAACAAATAGGGGTTCCGCGCACATTTCCCCGAAAAGTGCCACCTGACGTCTAAGAAACCATTATTATCATGACATTAACCTATAAAAATAGGCGTATCACGAGGCCCTTTCGTC |
| pAAV-CasRx-control presgRNA (pM123)- Plasmid DNA | CCTGCAGGCAGCTGCGCGCTCGCTCGCTCACTGAGGCCGCCCGGGCGTCGGGCGACCTTTGGTCGCCCGGCCTCAGTGAGCGAGCGAGCGCGCAGAGAGGGAGTGGCCAACTCCATCACTAGGGGTTCCTGCGGCCGCACGCGTGAGGGCCTATTTCCCATGATTCCTTCATATTTGCATATACGATACAAGGCTGTTAGAGAGATAATTGGAATTAATTTGACTGTAAACACAAAGATATTAGTACAAAATACGTGACGTAGAAAGTAATAATTTCTTGGGTAGTTTGCAGTTTTAAAATTATGTTTTAAAATGGACTATCATATGCTTACCGTAACTTGAAAGTATTTCGATTTCTTGGCTTTATATATCTTGTGGAAAGGACGAAACACCGCAAGTAAACCCCTACCAACTGGTCGGGGTTTGAAAC**TCACCAGAAGCGTACCATACTCACGAACAG**CAAGTAAACCCCTACCAACTGGTCGGGGTTTGAAACTTTTTTTGAATTCTGATGCGGTGAAGCTTTAGGTCTTGAAAGGAGTGGGAATTGGCTCCGGTGCCCGTCAGTGGGCAGAGCGCACATCGCCCACAGTCCCCGAGAAGTTGGGGGGAGGGGTCGGCAATTGATCCGGTGCCTAGAGAAGGTGGCGCGGGGTAAACTGGGAAAGTGATGTCGTGTACTGGCTCCGCCTTTTTCCCGAGGGTGGGGGAGAACCGTATATAAGTGCAGTAGTCGCCGTGAACGTTCTTTTTCGCAACGGGTTTGCCGCCAGAACACAGGTCTAGAGCCACCATGAGCCCCAAGAAGAAGAGAAAGGTGGAGGCCAGCATCGAAAAAAAAAAGTCCTTCGCCAAGGGCATGGGCGTGAAGTCCACACTCGTGTCCGGCTCCAAAGTGTACATGACAACCTTCGCCGAAGGCAGCGACGCCAGGCTGGAAAAGATCGTGGAGGGCGACAGCATCAGGAGCGTGAATGAGGGCGAGGCCTTCAGCGCTGAAATGGCCGATAAAAACGCCGGCTATAAGATCGGCAACGCCAAATTCAGCCATCCTAAGGGCTACGCCGTGGTGGCTAACAACCCTCTGTATACAGGACCCGTCCAGCAGGATATGCTCGGCCTGAAGGAAACTCTGGAAAAGAGGTACTTCGGCGAGAGCGCTGATGGCAATGACAATATTTGTATCCAGGTGATCCATAACATCCTGGACATTGAAAAAATCCTCGCCGAATACATTACCAACGCCGCCTACGCCGTCAACAATATCTCCGGCCTGGATAAGGACATTATTGGATTCGGCAAGTTCTCCACAGTGTATACCTACGACGAATTCAAAGACCCCGAGCACCATAGGGCCGCTTTCAACAATAACGATAAGCTCATCAACGCCATCAAGGCCCAGTATGACGAGTTCGACAACTTCCTCGATAACCCCAGACTCGGCTATTTCGGCCAGGCCTTTTTCAGCAAGGAGGGCAGAAATTACATCATCAATTACGGCAACGAATGCTATGACATTCTGGCCCTCCTGAGCGGACTGAGGCACTGGGTGGTCCATAACAACGAAGAAGAGTCCAGGATCTCCAGGACCTGGCTCTACAACCTCGATAAGAACCTCGACAACGAATACATCTCCACCCTCAACTACCTCTACGACAGGATCACCAATGAGCTGACCAACTCCTTCTCCAAGAACTCCGCCGCCAACGTGAACTATATTGCCGAAACTCTGGGAATCAACCCTGCCGAATTCGCCGAACAATATTTCAGATTCAGCATTATGAAAGAGCAGAAAAACCTCGGATTCAATATCACCAAGCTCAGGGAAGTGATGCTGGACAGGAAGGATATGTCCGAGATCAGGAAAAATCATAAGGTGTTCGACTCCATCAGGACCAAGGTCTACACCATGATGGACTTTGTGATTTATAGGTATTACATCGAAGAGGATGCCAAGGTGGCTGCCGCCAATAAGTCCCTCCCCGATAATGAGAAGTCCCTGAGCGAGAAGGATATCTTTGTGATTAACCTGAGGGGCTCCTTCAACGACGACCAGAAGGATGCCCTCTACTACGATGAAGCTAATAGAATTTGGAGAAAGCTCGAAAATATCATGCACAACATCAAGGAATTTAGGGGAAACAAGACAAGAGAGTATAAGAAGAAGGACGCCCCTAGACTGCCCAGAATCCTGCCCGCTGGCCGTGATGTTTCCGCCTTCAGCAAACTCATGTATGCCCTGACCATGTTCCTGGATGGCAAGGAGATCAACGACCTCCTGACCACCCTGATTAATAAATTCGATAACATCCAGAGCTTCCTGAAGGTGATGCCTCTCATCGGAGTCAACGCTAAGTTCGTGGAGGAATACGCCTTTTTCAAAGACTCCGCCAAGATCGCCGATGAGCTGAGGCTGATCAAGTCCTTCGCTAGAATGGGAGAACCTATTGCCGATGCCAGGAGGGCCATGTATATCGACGCCATCCGTATTTTAGGAACCAACCTGTCCTATGATGAGCTCAAGGCCCTCGCCGACACCTTTTCCCTGGACGAGAACGGAAACAAGCTCAAGAAAGGCAAGCACGGCATGAGAAATTTCATTATTAATAACGTGATCAGCAATAAAAGGTTCCACTACCTGATCAGATACGGTGATCCTGCCCACCTCCATGAGATCGCCAAAAACGAGGCCGTGGTGAAGTTCGTGCTCGGCAGGATCGCTGACATCCAGAAAAAACAGGGCCAGAACGGCAAGAACCAGATCGACAGGTACTACGAAACTTGTATCGGAAAGGATAAGGGCAAGAGCGTGAGCGAAAAGGTGGACGCTCTCACAAAGATCATCACCGGAATGAACTACGACCAATTCGACAAGAAAAGGAGCGTCATTGAGGACACCGGCAGGGAAAACGCCGAGAGGGAGAAGTTTAAAAAGATCATCAGCCTGTACCTCACCGTGATCTACCACATCCTCAAGAATATTGTCAATATCAACGCCAGGTACGTCATCGGATTCCATTGCGTCGAGCGTGATGCTCAACTGTACAAGGAGAAAGGCTACGACATCAATCTCAAGAAACTGGAAGAGAAGGGATTCAGCTCCGTCACCAAGCTCTGCGCTGGCATTGATGAAACTGCCCCCGATAAGAGAAAGGACGTGGAAAAGGAGATGGCTGAAAGAGCCAAGGAGAGCATTGACAGCCTCGAGAGCGCCAACCCCAAGCTGTATGCCAATTACATCAAATACAGCGACGAGAAGAAAGCCGAGGAGTTCACCAGGCAGATTAACAGGGAGAAGGCCAAAACCGCCCTGAACGCCTACCTGAGGAACACCAAGTGGAATGTGATCATCAGGGAGGACCTCCTGAGAATTGACAACAAGACATGTACCCTGTTCAGAAACAAGGCCGTCCACCTGGAAGTGGCCAGGTATGTCCACGCCTATATCAACGACATTGCCGAGGTCAATTCCTACTTCCAACTGTACCATTACATCATGCAGAGAATTATCATGAATGAGAGGTACGAGAAAAGCAGCGGAAAGGTGTCCGAGTACTTCGACGCTGTGAATGACGAGAAGAAGTACAACGATAGGCTCCTGAAACTGCTGTGTGTGCCTTTCGGCTACTGTATCCCCAGGTTTAAGAACCTGAGCATCGAGGCCCTGTTCGATAGGAACGAGGCCGCCAAGTTCGACAAGGAGAAAAAGAAGGTGTCCGGCAATTCCGGATCCGGACCTAAGAAAAAGAGGAAGGTGGCGGCCGCTTACCCATACGATGTTCCAGATTACGCTTAAGTCGACAACTTGTTTATTGCAGCTTATAATGGTTACAAATAAAGCAATAGCATCACAAATTTCACAAATAAAGCATTTTTTTCACTGCATTCTAGTTGTGGTTTGTCCAAACTCATCAATGTATCTTATCATGTCTGGATCCGGACCGAGCGGCCGCAGGAACCCCTAGTGATGGAGTTGGCCACTCCCTCTCTGCGCGCTCGCTCGCTCACTGAGGCCGGGCGACCAAAGGTCGCCCGACGCCCGGGCTTTGCCCGGGCGGCCTCAGTGAGCGAGCGAGCGCGCAGCTGCCTGCAGGGGCGCCTGATGCGGTATTTTCTCCTTACGCATCTGTGCGGTATTTCACACCGCATACGTCAAAGCAACCATAGTACGCGCCCTGTAGCGGCGCATTAAGCGCGGCGGGTGTGGTGGTTACGCGCAGCGTGACCGCTACACTTGCCAGCGCCCTAGCGCCCGCTCCTTTCGCTTTCTTCCCTTCCTTTCTCGCCACGTTCGCCGGCTTTCCCCGTCAAGCTCTAAATCGGGGGCTCCCTTTAGGGTTCCGATTTAGTGCTTTACGGCACCTCGACCCCAAAAAACTTGATTTGGGTGATGGTTCACGTAGTGGGCCATCGCCCTGATAGACGGTTTTTCGCCCTTTGACGTTGGAGTCCACGTTCTTTAATAGTGGACTCTTGTTCCAAACTGGAACAACACTCAACCCTATCTCGGGCTATTCTTTTGATTTATAAGGGATTTTGCCGATTTCGGCCTATTGGTTAAAAAATGAGCTGATTTAACAAAAATTTAACGCGAATTTTAACAAAATATTAACGTTTACAATTTTATGGTGCACTCTCAGTACAATCTGCTCTGATGCCGCATAGTTAAGCCAGCCCCGACACCCGCCAACACCCGCTGACGCGCCCTGACGGGCTTGTCTGCTCCCGGCATCCGCTTACAGACAAGCTGTGACCGTCTCCGGGAGCTGCATGTGTCAGAGGTTTTCACCGTCATCACCGAAACGCGCGAGACGAAAGGGCCTCGTGATACGCCTATTTTTATAGGTTAATGTCATGATAATAATGGTTTCTTAGACGTCAGGTGGCACTTTTCGGGGAAATGTGCGCGGAACCCCTATTTGTTTATTTTTCTAAATACATTCAAATATGTATCCGCTCATGAGACAATAACCCTGATAAATGCTTCAATAATATTGAAAAAGGAAGAGTATGAGTATTCAACATTTCCGTGTCGCCCTTATTCCCTTTTTTGCGGCATTTTGCCTTCCTGTTTTTGCTCACCCAGAAACGCTGGTGAAAGTAAAAGATGCTGAAGATCAGTTGGGTGCACGAGTGGGTTACATCGAACTGGATCTCAACAGCGGTAAGATCCTTGAGAGTTTTCGCCCCGAAGAACGTTTTCCAATGATGAGCACTTTTAAAGTTCTGCTATGTGGCGCGGTATTATCCCGTATTGACGCCGGGCAAGAGCAACTCGGTCGCCGCATACACTATTCTCAGAATGACTTGGTTGAGTACTCACCAGTCACAGAAAAGCATCTTACGGATGGCATGACAGTAAGAGAATTATGCAGTGCTGCCATAACCATGAGTGATAACACTGCGGCCAACTTACTTCTGACAACGATCGGAGGACCGAAGGAGCTAACCGCTTTTTTGCACAACATGGGGGATCATGTAACTCGCCTTGATCGTTGGGAACCGGAGCTGAATGAAGCCATACCAAACGACGAGCGTGACACCACGATGCCTGTAGCAATGGCAACAACGTTGCGCAAACTATTAACTGGCGAACTACTTACTCTAGCTTCCCGGCAACAATTAATAGACTGGATGGAGGCGGATAAAGTTGCAGGACCACTTCTGCGCTCGGCCCTTCCGGCTGGCTGGTTTATTGCTGATAAATCTGGAGCCGGTGAGCGTGGGTCTCGCGGTATCATTGCAGCACTGGGGCCAGATGGTAAGCCCTCCCGTATCGTAGTTATCTACACGACGGGGAGTCAGGCAACTATGGATGAACGAAATAGACAGATCGCTGAGATAGGTGCCTCACTGATTAAGCATTGGTAACTGTCAGACCAAGTTTACTCATATATACTTTAGATTGATTTAAAACTTCATTTTTAATTTAAAAGGATCTAGGTGAAGATCCTTTTTGATAATCTCATGACCAAAATCCCTTAACGTGAGTTTTCGTTCCACTGAGCGTCAGACCCCGTAGAAAAGATCAAAGGATCTTCTTGAGATCCTTTTTTTCTGCGCGTAATCTGCTGCTTGCAAACAAAAAAACCACCGCTACCAGCGGTGGTTTGTTTGCCGGATCAAGAGCTACCAACTCTTTTTCCGAAGGTAACTGGCTTCAGCAGAGCGCAGATACCAAATACTGTCCTTCTAGTGTAGCCGTAGTTAGGCCACCACTTCAAGAACTCTGTAGCACCGCCTACATACCTCGCTCTGCTAATCCTGTTACCAGTGGCTGCTGCCAGTGGCGATAAGTCGTGTCTTACCGGGTTGGACTCAAGACGATAGTTACCGGATAAGGCGCAGCGGTCGGGCTGAACGGGGGGTTCGTGCACACAGCCCAGCTTGGAGCGAACGACCTACACCGAACTGAGATACCTACAGCGTGAGCTATGAGAAAGCGCCACGCTTCCCGAAGGGAGAAAGGCGGACAGGTATCCGGTAAGCGGCAGGGTCGGAACAGGAGAGCGCACGAGGGAGCTTCCAGGGGGAAACGCCTGGTATCTTTATAGTCCTGTCGGGTTTCGCCACCTCTGACTTGAGCGTCGATTTTTGTGATGCTCGTCAGGGGGGCGGAGCCTATGGAAAAACGCCAGCAACGCGGCCTTTTTACGGTTCCTGGCCTTTTGCTGGCCTTTTGCTCACATGT |
| pAAV-CasRx-VEGFA presgRNA (pM124)- Plasmid DNA | CCTGCAGGCAGCTGCGCGCTCGCTCGCTCACTGAGGCCGCCCGGGCGTCGGGCGACCTTTGGTCGCCCGGCCTCAGTGAGCGAGCGAGCGCGCAGAGAGGGAGTGGCCAACTCCATCACTAGGGGTTCCTGCGGCCGCACGCGTGAGGGCCTATTTCCCATGATTCCTTCATATTTGCATATACGATACAAGGCTGTTAGAGAGATAATTGGAATTAATTTGACTGTAAACACAAAGATATTAGTACAAAATACGTGACGTAGAAAGTAATAATTTCTTGGGTAGTTTGCAGTTTTAAAATTATGTTTTAAAATGGACTATCATATGCTTACCGTAACTTGAAAGTATTTCGATTTCTTGGCTTTATATATCTTGTGGAAAGGACGAAACACCGCAAGTAAACCCCTACCAACTGGTCGGGGTTTGAAAC**TATGTGCTGGCCTTGGTGAGGTTTGATCCG**CAAGTAAACCCCTACCAACTGGTCGGGGTTTGAAACTTTTTTTGAATTCTGATGCGGTGAAGCTTTAGGTCTTGAAAGGAGTGGGAATTGGCTCCGGTGCCCGTCAGTGGGCAGAGCGCACATCGCCCACAGTCCCCGAGAAGTTGGGGGGAGGGGTCGGCAATTGATCCGGTGCCTAGAGAAGGTGGCGCGGGGTAAACTGGGAAAGTGATGTCGTGTACTGGCTCCGCCTTTTTCCCGAGGGTGGGGGAGAACCGTATATAAGTGCAGTAGTCGCCGTGAACGTTCTTTTTCGCAACGGGTTTGCCGCCAGAACACAGGTCTAGAGCCACCATGAGCCCCAAGAAGAAGAGAAAGGTGGAGGCCAGCATCGAAAAAAAAAAGTCCTTCGCCAAGGGCATGGGCGTGAAGTCCACACTCGTGTCCGGCTCCAAAGTGTACATGACAACCTTCGCCGAAGGCAGCGACGCCAGGCTGGAAAAGATCGTGGAGGGCGACAGCATCAGGAGCGTGAATGAGGGCGAGGCCTTCAGCGCTGAAATGGCCGATAAAAACGCCGGCTATAAGATCGGCAACGCCAAATTCAGCCATCCTAAGGGCTACGCCGTGGTGGCTAACAACCCTCTGTATACAGGACCCGTCCAGCAGGATATGCTCGGCCTGAAGGAAACTCTGGAAAAGAGGTACTTCGGCGAGAGCGCTGATGGCAATGACAATATTTGTATCCAGGTGATCCATAACATCCTGGACATTGAAAAAATCCTCGCCGAATACATTACCAACGCCGCCTACGCCGTCAACAATATCTCCGGCCTGGATAAGGACATTATTGGATTCGGCAAGTTCTCCACAGTGTATACCTACGACGAATTCAAAGACCCCGAGCACCATAGGGCCGCTTTCAACAATAACGATAAGCTCATCAACGCCATCAAGGCCCAGTATGACGAGTTCGACAACTTCCTCGATAACCCCAGACTCGGCTATTTCGGCCAGGCCTTTTTCAGCAAGGAGGGCAGAAATTACATCATCAATTACGGCAACGAATGCTATGACATTCTGGCCCTCCTGAGCGGACTGAGGCACTGGGTGGTCCATAACAACGAAGAAGAGTCCAGGATCTCCAGGACCTGGCTCTACAACCTCGATAAGAACCTCGACAACGAATACATCTCCACCCTCAACTACCTCTACGACAGGATCACCAATGAGCTGACCAACTCCTTCTCCAAGAACTCCGCCGCCAACGTGAACTATATTGCCGAAACTCTGGGAATCAACCCTGCCGAATTCGCCGAACAATATTTCAGATTCAGCATTATGAAAGAGCAGAAAAACCTCGGATTCAATATCACCAAGCTCAGGGAAGTGATGCTGGACAGGAAGGATATGTCCGAGATCAGGAAAAATCATAAGGTGTTCGACTCCATCAGGACCAAGGTCTACACCATGATGGACTTTGTGATTTATAGGTATTACATCGAAGAGGATGCCAAGGTGGCTGCCGCCAATAAGTCCCTCCCCGATAATGAGAAGTCCCTGAGCGAGAAGGATATCTTTGTGATTAACCTGAGGGGCTCCTTCAACGACGACCAGAAGGATGCCCTCTACTACGATGAAGCTAATAGAATTTGGAGAAAGCTCGAAAATATCATGCACAACATCAAGGAATTTAGGGGAAACAAGACAAGAGAGTATAAGAAGAAGGACGCCCCTAGACTGCCCAGAATCCTGCCCGCTGGCCGTGATGTTTCCGCCTTCAGCAAACTCATGTATGCCCTGACCATGTTCCTGGATGGCAAGGAGATCAACGACCTCCTGACCACCCTGATTAATAAATTCGATAACATCCAGAGCTTCCTGAAGGTGATGCCTCTCATCGGAGTCAACGCTAAGTTCGTGGAGGAATACGCCTTTTTCAAAGACTCCGCCAAGATCGCCGATGAGCTGAGGCTGATCAAGTCCTTCGCTAGAATGGGAGAACCTATTGCCGATGCCAGGAGGGCCATGTATATCGACGCCATCCGTATTTTAGGAACCAACCTGTCCTATGATGAGCTCAAGGCCCTCGCCGACACCTTTTCCCTGGACGAGAACGGAAACAAGCTCAAGAAAGGCAAGCACGGCATGAGAAATTTCATTATTAATAACGTGATCAGCAATAAAAGGTTCCACTACCTGATCAGATACGGTGATCCTGCCCACCTCCATGAGATCGCCAAAAACGAGGCCGTGGTGAAGTTCGTGCTCGGCAGGATCGCTGACATCCAGAAAAAACAGGGCCAGAACGGCAAGAACCAGATCGACAGGTACTACGAAACTTGTATCGGAAAGGATAAGGGCAAGAGCGTGAGCGAAAAGGTGGACGCTCTCACAAAGATCATCACCGGAATGAACTACGACCAATTCGACAAGAAAAGGAGCGTCATTGAGGACACCGGCAGGGAAAACGCCGAGAGGGAGAAGTTTAAAAAGATCATCAGCCTGTACCTCACCGTGATCTACCACATCCTCAAGAATATTGTCAATATCAACGCCAGGTACGTCATCGGATTCCATTGCGTCGAGCGTGATGCTCAACTGTACAAGGAGAAAGGCTACGACATCAATCTCAAGAAACTGGAAGAGAAGGGATTCAGCTCCGTCACCAAGCTCTGCGCTGGCATTGATGAAACTGCCCCCGATAAGAGAAAGGACGTGGAAAAGGAGATGGCTGAAAGAGCCAAGGAGAGCATTGACAGCCTCGAGAGCGCCAACCCCAAGCTGTATGCCAATTACATCAAATACAGCGACGAGAAGAAAGCCGAGGAGTTCACCAGGCAGATTAACAGGGAGAAGGCCAAAACCGCCCTGAACGCCTACCTGAGGAACACCAAGTGGAATGTGATCATCAGGGAGGACCTCCTGAGAATTGACAACAAGACATGTACCCTGTTCAGAAACAAGGCCGTCCACCTGGAAGTGGCCAGGTATGTCCACGCCTATATCAACGACATTGCCGAGGTCAATTCCTACTTCCAACTGTACCATTACATCATGCAGAGAATTATCATGAATGAGAGGTACGAGAAAAGCAGCGGAAAGGTGTCCGAGTACTTCGACGCTGTGAATGACGAGAAGAAGTACAACGATAGGCTCCTGAAACTGCTGTGTGTGCCTTTCGGCTACTGTATCCCCAGGTTTAAGAACCTGAGCATCGAGGCCCTGTTCGATAGGAACGAGGCCGCCAAGTTCGACAAGGAGAAAAAGAAGGTGTCCGGCAATTCCGGATCCGGACCTAAGAAAAAGAGGAAGGTGGCGGCCGCTTACCCATACGATGTTCCAGATTACGCTTAAGTCGACAACTTGTTTATTGCAGCTTATAATGGTTACAAATAAAGCAATAGCATCACAAATTTCACAAATAAAGCATTTTTTTCACTGCATTCTAGTTGTGGTTTGTCCAAACTCATCAATGTATCTTATCATGTCTGGATCCGGACCGAGCGGCCGCAGGAACCCCTAGTGATGGAGTTGGCCACTCCCTCTCTGCGCGCTCGCTCGCTCACTGAGGCCGGGCGACCAAAGGTCGCCCGACGCCCGGGCTTTGCCCGGGCGGCCTCAGTGAGCGAGCGAGCGCGCAGCTGCCTGCAGGGGCGCCTGATGCGGTATTTTCTCCTTACGCATCTGTGCGGTATTTCACACCGCATACGTCAAAGCAACCATAGTACGCGCCCTGTAGCGGCGCATTAAGCGCGGCGGGTGTGGTGGTTACGCGCAGCGTGACCGCTACACTTGCCAGCGCCCTAGCGCCCGCTCCTTTCGCTTTCTTCCCTTCCTTTCTCGCCACGTTCGCCGGCTTTCCCCGTCAAGCTCTAAATCGGGGGCTCCCTTTAGGGTTCCGATTTAGTGCTTTACGGCACCTCGACCCCAAAAAACTTGATTTGGGTGATGGTTCACGTAGTGGGCCATCGCCCTGATAGACGGTTTTTCGCCCTTTGACGTTGGAGTCCACGTTCTTTAATAGTGGACTCTTGTTCCAAACTGGAACAACACTCAACCCTATCTCGGGCTATTCTTTTGATTTATAAGGGATTTTGCCGATTTCGGCCTATTGGTTAAAAAATGAGCTGATTTAACAAAAATTTAACGCGAATTTTAACAAAATATTAACGTTTACAATTTTATGGTGCACTCTCAGTACAATCTGCTCTGATGCCGCATAGTTAAGCCAGCCCCGACACCCGCCAACACCCGCTGACGCGCCCTGACGGGCTTGTCTGCTCCCGGCATCCGCTTACAGACAAGCTGTGACCGTCTCCGGGAGCTGCATGTGTCAGAGGTTTTCACCGTCATCACCGAAACGCGCGAGACGAAAGGGCCTCGTGATACGCCTATTTTTATAGGTTAATGTCATGATAATAATGGTTTCTTAGACGTCAGGTGGCACTTTTCGGGGAAATGTGCGCGGAACCCCTATTTGTTTATTTTTCTAAATACATTCAAATATGTATCCGCTCATGAGACAATAACCCTGATAAATGCTTCAATAATATTGAAAAAGGAAGAGTATGAGTATTCAACATTTCCGTGTCGCCCTTATTCCCTTTTTTGCGGCATTTTGCCTTCCTGTTTTTGCTCACCCAGAAACGCTGGTGAAAGTAAAAGATGCTGAAGATCAGTTGGGTGCACGAGTGGGTTACATCGAACTGGATCTCAACAGCGGTAAGATCCTTGAGAGTTTTCGCCCCGAAGAACGTTTTCCAATGATGAGCACTTTTAAAGTTCTGCTATGTGGCGCGGTATTATCCCGTATTGACGCCGGGCAAGAGCAACTCGGTCGCCGCATACACTATTCTCAGAATGACTTGGTTGAGTACTCACCAGTCACAGAAAAGCATCTTACGGATGGCATGACAGTAAGAGAATTATGCAGTGCTGCCATAACCATGAGTGATAACACTGCGGCCAACTTACTTCTGACAACGATCGGAGGACCGAAGGAGCTAACCGCTTTTTTGCACAACATGGGGGATCATGTAACTCGCCTTGATCGTTGGGAACCGGAGCTGAATGAAGCCATACCAAACGACGAGCGTGACACCACGATGCCTGTAGCAATGGCAACAACGTTGCGCAAACTATTAACTGGCGAACTACTTACTCTAGCTTCCCGGCAACAATTAATAGACTGGATGGAGGCGGATAAAGTTGCAGGACCACTTCTGCGCTCGGCCCTTCCGGCTGGCTGGTTTATTGCTGATAAATCTGGAGCCGGTGAGCGTGGGTCTCGCGGTATCATTGCAGCACTGGGGCCAGATGGTAAGCCCTCCCGTATCGTAGTTATCTACACGACGGGGAGTCAGGCAACTATGGATGAACGAAATAGACAGATCGCTGAGATAGGTGCCTCACTGATTAAGCATTGGTAACTGTCAGACCAAGTTTACTCATATATACTTTAGATTGATTTAAAACTTCATTTTTAATTTAAAAGGATCTAGGTGAAGATCCTTTTTGATAATCTCATGACCAAAATCCCTTAACGTGAGTTTTCGTTCCACTGAGCGTCAGACCCCGTAGAAAAGATCAAAGGATCTTCTTGAGATCCTTTTTTTCTGCGCGTAATCTGCTGCTTGCAAACAAAAAAACCACCGCTACCAGCGGTGGTTTGTTTGCCGGATCAAGAGCTACCAACTCTTTTTCCGAAGGTAACTGGCTTCAGCAGAGCGCAGATACCAAATACTGTCCTTCTAGTGTAGCCGTAGTTAGGCCACCACTTCAAGAACTCTGTAGCACCGCCTACATACCTCGCTCTGCTAATCCTGTTACCAGTGGCTGCTGCCAGTGGCGATAAGTCGTGTCTTACCGGGTTGGACTCAAGACGATAGTTACCGGATAAGGCGCAGCGGTCGGGCTGAACGGGGGGTTCGTGCACACAGCCCAGCTTGGAGCGAACGACCTACACCGAACTGAGATACCTACAGCGTGAGCTATGAGAAAGCGCCACGCTTCCCGAAGGGAGAAAGGCGGACAGGTATCCGGTAAGCGGCAGGGTCGGAACAGGAGAGCGCACGAGGGAGCTTCCAGGGGGAAACGCCTGGTATCTTTATAGTCCTGTCGGGTTTCGCCACCTCTGACTTGAGCGTCGATTTTTGTGATGCTCGTCAGGGGGGCGGAGCCTATGGAAAAACGCCAGCAACGCGGCCTTTTTACGGTTCCTGGCCTTTTGCTGGCCTTTTGCTCACATGT |
| pAAV-CasRx-VEGFA presgRNA array (pM125)- Plasmid DNA | CCTGCAGGCAGCTGCGCGCTCGCTCGCTCACTGAGGCCGCCCGGGCGTCGGGCGACCTTTGGTCGCCCGGCCTCAGTGAGCGAGCGAGCGCGCAGAGAGGGAGTGGCCAACTCCATCACTAGGGGTTCCTGCGGCCGCACGCGTGAGGGCCTATTTCCCATGATTCCTTCATATTTGCATATACGATACAAGGCTGTTAGAGAGATAATTGGAATTAATTTGACTGTAAACACAAAGATATTAGTACAAAATACGTGACGTAGAAAGTAATAATTTCTTGGGTAGTTTGCAGTTTTAAAATTATGTTTTAAAATGGACTATCATATGCTTACCGTAACTTGAAAGTATTTCGATTTCTTGGCTTTATATATCTTGTGGAAAGGACGAAACACCGCAAGTAAACCCCTACCAACTGGTCGGGGTTTGAAAC**TATGTGCTGGCCTTGGTGAGGTTTGATCCG**CAAGTAAACCCCTACCAACTGGTCGGGGTTTGAAAC**CACTTCGTGATGATTCTGCCCTCCTCCTTC**CAAGTAAACCCCTACCAACTGGTCGGGGTTTGAAAC**TCTGCATTCACATTTGTTGTGCTGTAGGAA**CAAGTAAACCCCTACCAACTGGTCGGGGTTTGAAACTTTTTTTGAATTCTGATGCGGTGAAGCTTTAGGTCTTGAAAGGAGTGGGAATTGGCTCCGGTGCCCGTCAGTGGGCAGAGCGCACATCGCCCACAGTCCCCGAGAAGTTGGGGGGAGGGGTCGGCAATTGATCCGGTGCCTAGAGAAGGTGGCGCGGGGTAAACTGGGAAAGTGATGTCGTGTACTGGCTCCGCCTTTTTCCCGAGGGTGGGGGAGAACCGTATATAAGTGCAGTAGTCGCCGTGAACGTTCTTTTTCGCAACGGGTTTGCCGCCAGAACACAGGTCTAGAGCCACCATGAGCCCCAAGAAGAAGAGAAAGGTGGAGGCCAGCATCGAAAAAAAAAAGTCCTTCGCCAAGGGCATGGGCGTGAAGTCCACACTCGTGTCCGGCTCCAAAGTGTACATGACAACCTTCGCCGAAGGCAGCGACGCCAGGCTGGAAAAGATCGTGGAGGGCGACAGCATCAGGAGCGTGAATGAGGGCGAGGCCTTCAGCGCTGAAATGGCCGATAAAAACGCCGGCTATAAGATCGGCAACGCCAAATTCAGCCATCCTAAGGGCTACGCCGTGGTGGCTAACAACCCTCTGTATACAGGACCCGTCCAGCAGGATATGCTCGGCCTGAAGGAAACTCTGGAAAAGAGGTACTTCGGCGAGAGCGCTGATGGCAATGACAATATTTGTATCCAGGTGATCCATAACATCCTGGACATTGAAAAAATCCTCGCCGAATACATTACCAACGCCGCCTACGCCGTCAACAATATCTCCGGCCTGGATAAGGACATTATTGGATTCGGCAAGTTCTCCACAGTGTATACCTACGACGAATTCAAAGACCCCGAGCACCATAGGGCCGCTTTCAACAATAACGATAAGCTCATCAACGCCATCAAGGCCCAGTATGACGAGTTCGACAACTTCCTCGATAACCCCAGACTCGGCTATTTCGGCCAGGCCTTTTTCAGCAAGGAGGGCAGAAATTACATCATCAATTACGGCAACGAATGCTATGACATTCTGGCCCTCCTGAGCGGACTGAGGCACTGGGTGGTCCATAACAACGAAGAAGAGTCCAGGATCTCCAGGACCTGGCTCTACAACCTCGATAAGAACCTCGACAACGAATACATCTCCACCCTCAACTACCTCTACGACAGGATCACCAATGAGCTGACCAACTCCTTCTCCAAGAACTCCGCCGCCAACGTGAACTATATTGCCGAAACTCTGGGAATCAACCCTGCCGAATTCGCCGAACAATATTTCAGATTCAGCATTATGAAAGAGCAGAAAAACCTCGGATTCAATATCACCAAGCTCAGGGAAGTGATGCTGGACAGGAAGGATATGTCCGAGATCAGGAAAAATCATAAGGTGTTCGACTCCATCAGGACCAAGGTCTACACCATGATGGACTTTGTGATTTATAGGTATTACATCGAAGAGGATGCCAAGGTGGCTGCCGCCAATAAGTCCCTCCCCGATAATGAGAAGTCCCTGAGCGAGAAGGATATCTTTGTGATTAACCTGAGGGGCTCCTTCAACGACGACCAGAAGGATGCCCTCTACTACGATGAAGCTAATAGAATTTGGAGAAAGCTCGAAAATATCATGCACAACATCAAGGAATTTAGGGGAAACAAGACAAGAGAGTATAAGAAGAAGGACGCCCCTAGACTGCCCAGAATCCTGCCCGCTGGCCGTGATGTTTCCGCCTTCAGCAAACTCATGTATGCCCTGACCATGTTCCTGGATGGCAAGGAGATCAACGACCTCCTGACCACCCTGATTAATAAATTCGATAACATCCAGAGCTTCCTGAAGGTGATGCCTCTCATCGGAGTCAACGCTAAGTTCGTGGAGGAATACGCCTTTTTCAAAGACTCCGCCAAGATCGCCGATGAGCTGAGGCTGATCAAGTCCTTCGCTAGAATGGGAGAACCTATTGCCGATGCCAGGAGGGCCATGTATATCGACGCCATCCGTATTTTAGGAACCAACCTGTCCTATGATGAGCTCAAGGCCCTCGCCGACACCTTTTCCCTGGACGAGAACGGAAACAAGCTCAAGAAAGGCAAGCACGGCATGAGAAATTTCATTATTAATAACGTGATCAGCAATAAAAGGTTCCACTACCTGATCAGATACGGTGATCCTGCCCACCTCCATGAGATCGCCAAAAACGAGGCCGTGGTGAAGTTCGTGCTCGGCAGGATCGCTGACATCCAGAAAAAACAGGGCCAGAACGGCAAGAACCAGATCGACAGGTACTACGAAACTTGTATCGGAAAGGATAAGGGCAAGAGCGTGAGCGAAAAGGTGGACGCTCTCACAAAGATCATCACCGGAATGAACTACGACCAATTCGACAAGAAAAGGAGCGTCATTGAGGACACCGGCAGGGAAAACGCCGAGAGGGAGAAGTTTAAAAAGATCATCAGCCTGTACCTCACCGTGATCTACCACATCCTCAAGAATATTGTCAATATCAACGCCAGGTACGTCATCGGATTCCATTGCGTCGAGCGTGATGCTCAACTGTACAAGGAGAAAGGCTACGACATCAATCTCAAGAAACTGGAAGAGAAGGGATTCAGCTCCGTCACCAAGCTCTGCGCTGGCATTGATGAAACTGCCCCCGATAAGAGAAAGGACGTGGAAAAGGAGATGGCTGAAAGAGCCAAGGAGAGCATTGACAGCCTCGAGAGCGCCAACCCCAAGCTGTATGCCAATTACATCAAATACAGCGACGAGAAGAAAGCCGAGGAGTTCACCAGGCAGATTAACAGGGAGAAGGCCAAAACCGCCCTGAACGCCTACCTGAGGAACACCAAGTGGAATGTGATCATCAGGGAGGACCTCCTGAGAATTGACAACAAGACATGTACCCTGTTCAGAAACAAGGCCGTCCACCTGGAAGTGGCCAGGTATGTCCACGCCTATATCAACGACATTGCCGAGGTCAATTCCTACTTCCAACTGTACCATTACATCATGCAGAGAATTATCATGAATGAGAGGTACGAGAAAAGCAGCGGAAAGGTGTCCGAGTACTTCGACGCTGTGAATGACGAGAAGAAGTACAACGATAGGCTCCTGAAACTGCTGTGTGTGCCTTTCGGCTACTGTATCCCCAGGTTTAAGAACCTGAGCATCGAGGCCCTGTTCGATAGGAACGAGGCCGCCAAGTTCGACAAGGAGAAAAAGAAGGTGTCCGGCAATTCCGGATCCGGACCTAAGAAAAAGAGGAAGGTGGCGGCCGCTTACCCATACGATGTTCCAGATTACGCTTAAGTCGACAACTTGTTTATTGCAGCTTATAATGGTTACAAATAAAGCAATAGCATCACAAATTTCACAAATAAAGCATTTTTTTCACTGCATTCTAGTTGTGGTTTGTCCAAACTCATCAATGTATCTTATCATGTCTGGATCCGGACCGAGCGGCCGCAGGAACCCCTAGTGATGGAGTTGGCCACTCCCTCTCTGCGCGCTCGCTCGCTCACTGAGGCCGGGCGACCAAAGGTCGCCCGACGCCCGGGCTTTGCCCGGGCGGCCTCAGTGAGCGAGCGAGCGCGCAGCTGCCTGCAGGGGCGCCTGATGCGGTATTTTCTCCTTACGCATCTGTGCGGTATTTCACACCGCATACGTCAAAGCAACCATAGTACGCGCCCTGTAGCGGCGCATTAAGCGCGGCGGGTGTGGTGGTTACGCGCAGCGTGACCGCTACACTTGCCAGCGCCCTAGCGCCCGCTCCTTTCGCTTTCTTCCCTTCCTTTCTCGCCACGTTCGCCGGCTTTCCCCGTCAAGCTCTAAATCGGGGGCTCCCTTTAGGGTTCCGATTTAGTGCTTTACGGCACCTCGACCCCAAAAAACTTGATTTGGGTGATGGTTCACGTAGTGGGCCATCGCCCTGATAGACGGTTTTTCGCCCTTTGACGTTGGAGTCCACGTTCTTTAATAGTGGACTCTTGTTCCAAACTGGAACAACACTCAACCCTATCTCGGGCTATTCTTTTGATTTATAAGGGATTTTGCCGATTTCGGCCTATTGGTTAAAAAATGAGCTGATTTAACAAAAATTTAACGCGAATTTTAACAAAATATTAACGTTTACAATTTTATGGTGCACTCTCAGTACAATCTGCTCTGATGCCGCATAGTTAAGCCAGCCCCGACACCCGCCAACACCCGCTGACGCGCCCTGACGGGCTTGTCTGCTCCCGGCATCCGCTTACAGACAAGCTGTGACCGTCTCCGGGAGCTGCATGTGTCAGAGGTTTTCACCGTCATCACCGAAACGCGCGAGACGAAAGGGCCTCGTGATACGCCTATTTTTATAGGTTAATGTCATGATAATAATGGTTTCTTAGACGTCAGGTGGCACTTTTCGGGGAAATGTGCGCGGAACCCCTATTTGTTTATTTTTCTAAATACATTCAAATATGTATCCGCTCATGAGACAATAACCCTGATAAATGCTTCAATAATATTGAAAAAGGAAGAGTATGAGTATTCAACATTTCCGTGTCGCCCTTATTCCCTTTTTTGCGGCATTTTGCCTTCCTGTTTTTGCTCACCCAGAAACGCTGGTGAAAGTAAAAGATGCTGAAGATCAGTTGGGTGCACGAGTGGGTTACATCGAACTGGATCTCAACAGCGGTAAGATCCTTGAGAGTTTTCGCCCCGAAGAACGTTTTCCAATGATGAGCACTTTTAAAGTTCTGCTATGTGGCGCGGTATTATCCCGTATTGACGCCGGGCAAGAGCAACTCGGTCGCCGCATACACTATTCTCAGAATGACTTGGTTGAGTACTCACCAGTCACAGAAAAGCATCTTACGGATGGCATGACAGTAAGAGAATTATGCAGTGCTGCCATAACCATGAGTGATAACACTGCGGCCAACTTACTTCTGACAACGATCGGAGGACCGAAGGAGCTAACCGCTTTTTTGCACAACATGGGGGATCATGTAACTCGCCTTGATCGTTGGGAACCGGAGCTGAATGAAGCCATACCAAACGACGAGCGTGACACCACGATGCCTGTAGCAATGGCAACAACGTTGCGCAAACTATTAACTGGCGAACTACTTACTCTAGCTTCCCGGCAACAATTAATAGACTGGATGGAGGCGGATAAAGTTGCAGGACCACTTCTGCGCTCGGCCCTTCCGGCTGGCTGGTTTATTGCTGATAAATCTGGAGCCGGTGAGCGTGGGTCTCGCGGTATCATTGCAGCACTGGGGCCAGATGGTAAGCCCTCCCGTATCGTAGTTATCTACACGACGGGGAGTCAGGCAACTATGGATGAACGAAATAGACAGATCGCTGAGATAGGTGCCTCACTGATTAAGCATTGGTAACTGTCAGACCAAGTTTACTCATATATACTTTAGATTGATTTAAAACTTCATTTTTAATTTAAAAGGATCTAGGTGAAGATCCTTTTTGATAATCTCATGACCAAAATCCCTTAACGTGAGTTTTCGTTCCACTGAGCGTCAGACCCCGTAGAAAAGATCAAAGGATCTTCTTGAGATCCTTTTTTTCTGCGCGTAATCTGCTGCTTGCAAACAAAAAAACCACCGCTACCAGCGGTGGTTTGTTTGCCGGATCAAGAGCTACCAACTCTTTTTCCGAAGGTAACTGGCTTCAGCAGAGCGCAGATACCAAATACTGTCCTTCTAGTGTAGCCGTAGTTAGGCCACCACTTCAAGAACTCTGTAGCACCGCCTACATACCTCGCTCTGCTAATCCTGTTACCAGTGGCTGCTGCCAGTGGCGATAAGTCGTGTCTTACCGGGTTGGACTCAAGACGATAGTTACCGGATAAGGCGCAGCGGTCGGGCTGAACGGGGGGTTCGTGCACACAGCCCAGCTTGGAGCGAACGACCTACACCGAACTGAGATACCTACAGCGTGAGCTATGAGAAAGCGCCACGCTTCCCGAAGGGAGAAAGGCGGACAGGTATCCGGTAAGCGGCAGGGTCGGAACAGGAGAGCGCACGAGGGAGCTTCCAGGGGGAAACGCCTGGTATCTTTATAGTCCTGTCGGGTTTCGCCACCTCTGACTTGAGCGTCGATTTTTGTGATGCTCGTCAGGGGGGCGGAGCCTATGGAAAAACGCCAGCAACGCGGCCTTTTTACGGTTCCTGGCCTTTTGCTGGCCTTTTGCTCACATGT |

**Table S2** List of plasmids and restriction enzymes used for plasmid validation with buffer used for respective restriction enzymes.

| **Description** | **Restriction Enzymes** | **Buffer** |
| --- | --- | --- |
| VEGFA shRNA | AhdI (R0584) and XmnI (R0194) | CutSmart^®^ |
| EF1α-CasRx-2A-EGFP | EcoRI-HF^®^ (R3101) | CutSmart^®^ |
| Control (non-targeting) presgRNA | AhdI (R0584) and XmnI (R0194) | CutSmart^®^ |
| VEGFA presgRNA | AhdI (R0584) and XmnI (R0194) | CutSmart^®^ |
| VEGFA presgRNA array | AhdI (R0584) and XmnI (R0194) | CutSmart^®^ |
| pAAV-CasRx-control presgRNA | SmaI (R0141) | CutSmart^®^ |
| pAAV-CasRx-VEGFA presgRNA | SmaI (R0141) | CutSmart^®^ |
| pAAV-CasRx-VEGFA presgRNA array | SmaI (R0141) | CutSmart^®^ |
