# Supplementary material for: Methods for in vitro CRISPR/CasRx-Mediated RNA Editing
Source: Front Cell Dev Biol. 2021 Jun 11;9:667879. doi: 10.3389/fcell.2021.667879 (PMC8226256; doi:10.3389/fcell.2021.667879)
Supplement: Supplementary file 2 [file Data_Sheet_1.PDF]

Title Page

Methods for *in vitro* CRISPR/CasRx-mediated RNA editing

Yu-Fan Chuang[1,2], Peng-Yuan Wang[1*], Satheesh Kumar S[2], Suraj Lama[2], Fan-Li Lin[1,2], Guei-Sheung Liu[2,3,4*]

[1]Shenzhen Key Laboratory of Biomimetic Materials and Cellular Immunomodulation, Shenzhen Institute of Advanced Technology, Chinese Academy of Sciences, Shenzhen, China
[2]Menzies Institute for Medical Research, University of Tasmania, Hobart, Australia
[3]Ophthalmology, Department of Surgery, University of Melbourne, East Melbourne, Victoria, Australia
[4]Aier Eye Institute, Changsha, Hunan, China

*Correspondence and requests for materials should be addressed to
Dr Guei-Sheung Liu (rickliu0817@gmail.com). Menzies Institute for Medical Research, University of Tasmania. Address: 17 Liverpool Street, Hobart, TAS 7000, Australia. Tel: +61362264250.
or
Dr Peng-Yuan Wang (py.wang@siat.ac.cn). Shenzhen Key Laboratory of Biomimetic Materials and Cellular Immunomodulation, Shenzhen Institute of Advanced Technology, Chinese Academy of Sciences. Address: 1068 Xueyuan Avenue, Shenzhen University Town, Shenzhen, China 518055. Tel: +86 755 8639 2694

Running title: Targeting VEGFA by CRISPR/CasRx RNA editing

SUPPLEMENTARY FIGURES
FIG. S1-S12

Figure S1. Human VEGFA cDNA and targeting sequence for sgRNA or presgRNA. Created with Benchling.

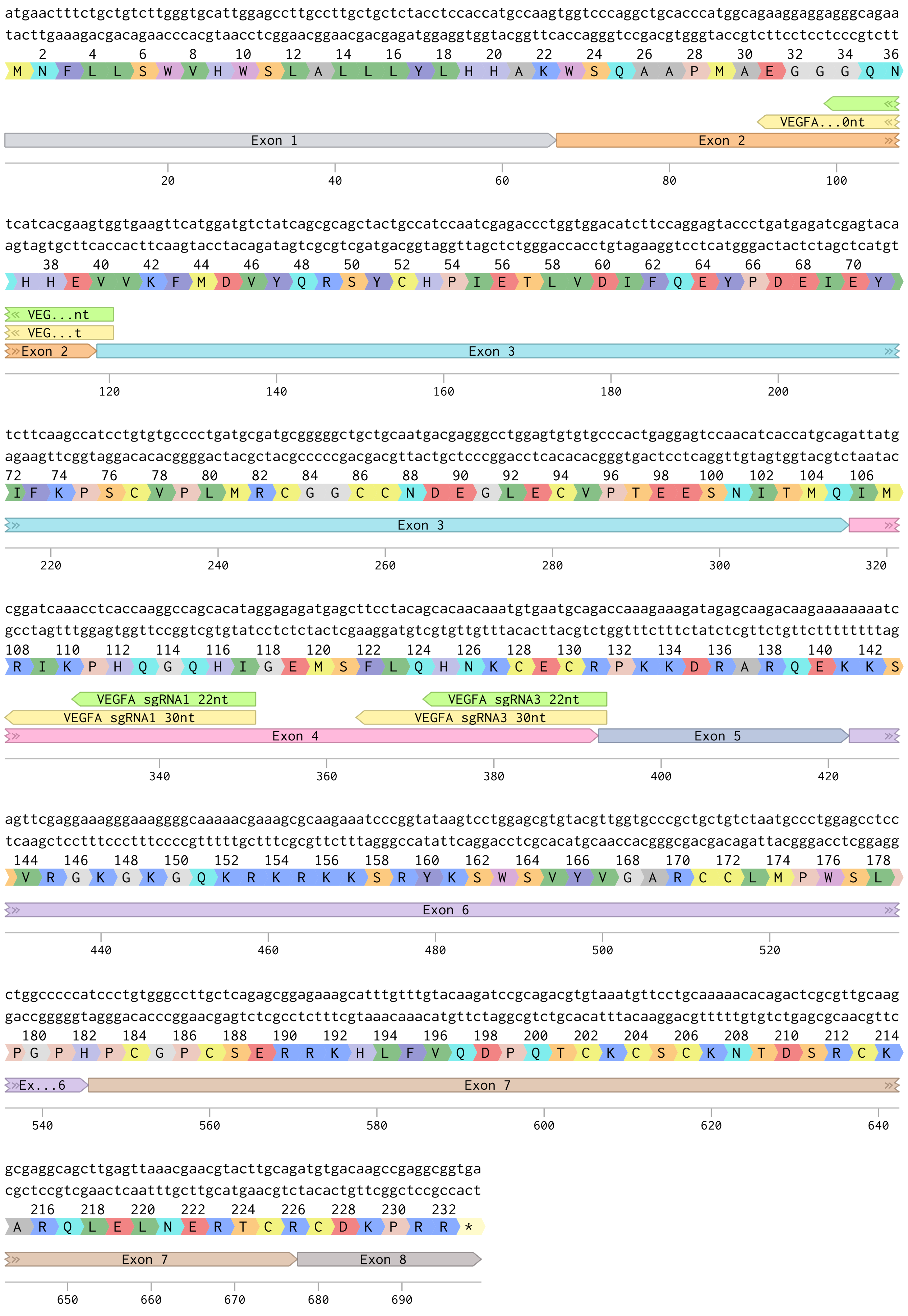

Figure S2. CasRx sgRNA expression cassette (427bp). Created with Benchling.

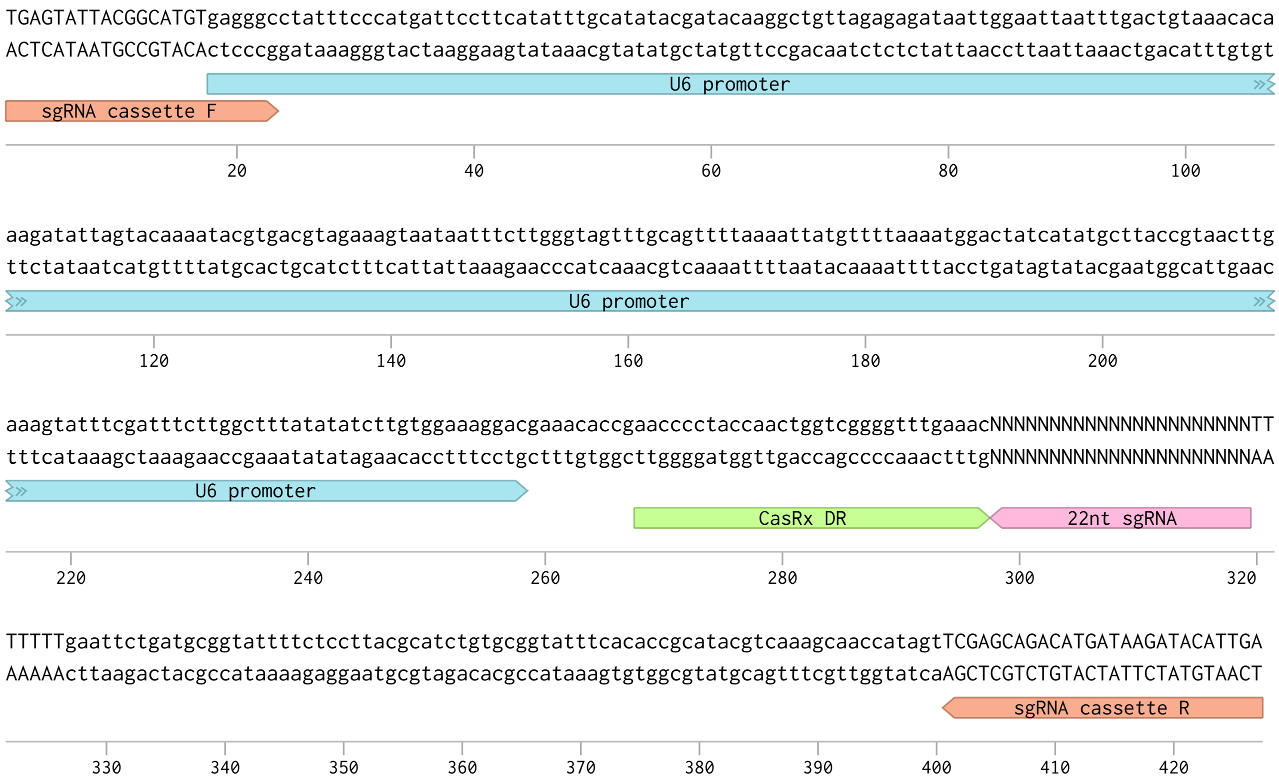

Figure S3. CasRx presgRNA cloning backbone. Created with Benchling.

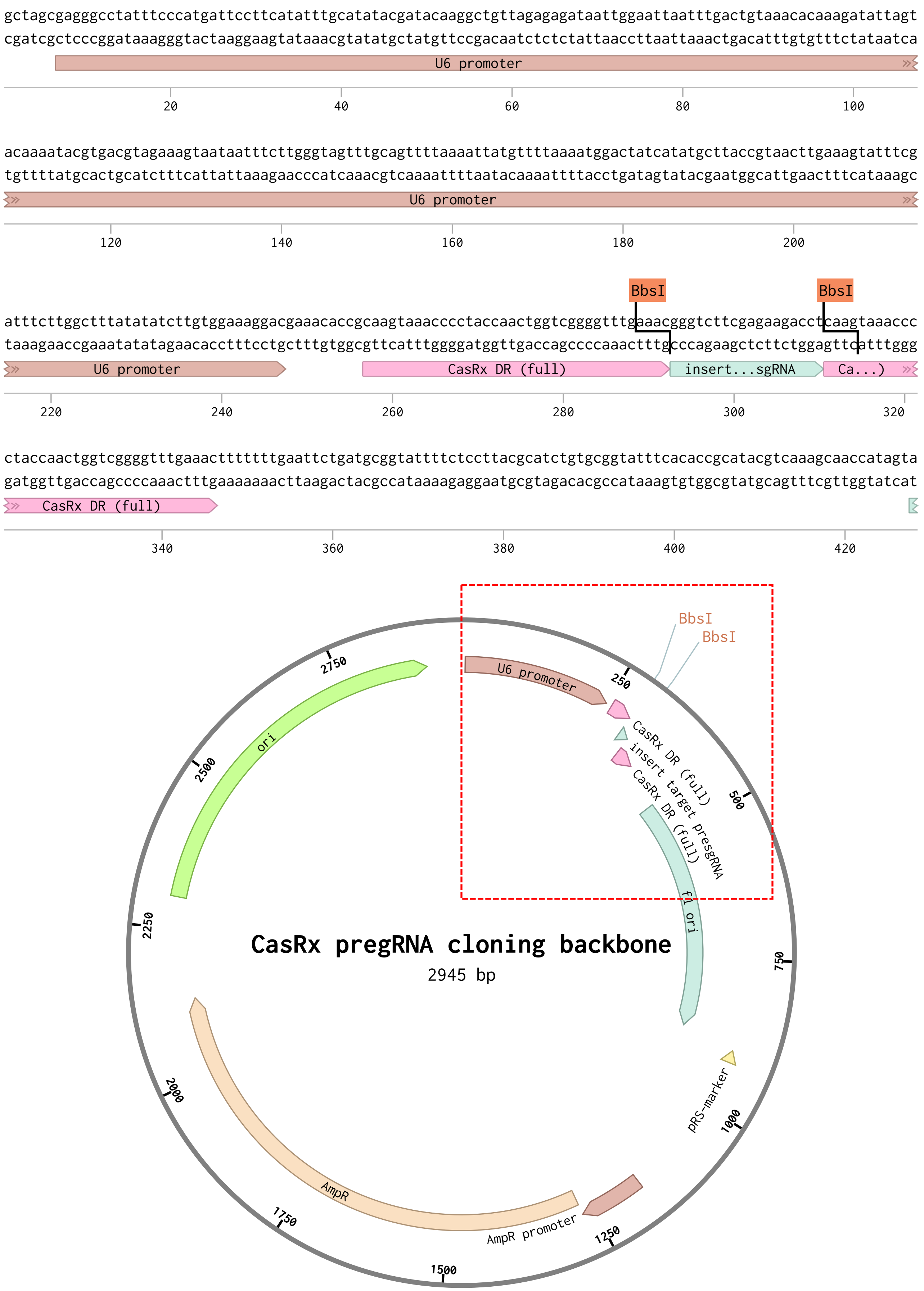

Figure S4. CasRx presgRNA expression cassette (419 bp). Created with Benchling.

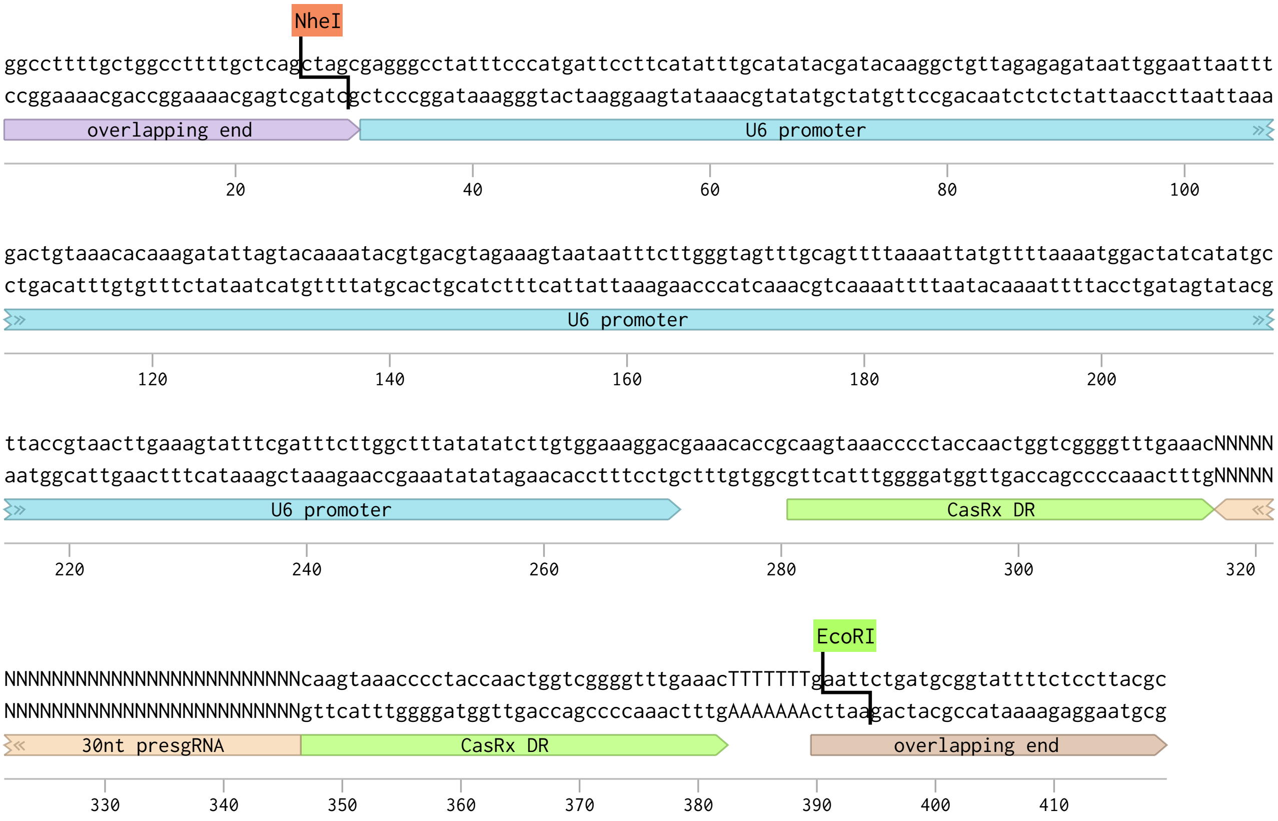

Figure S5. CasRx presgRNA array expression cassette (551bp). Created with Benchling.

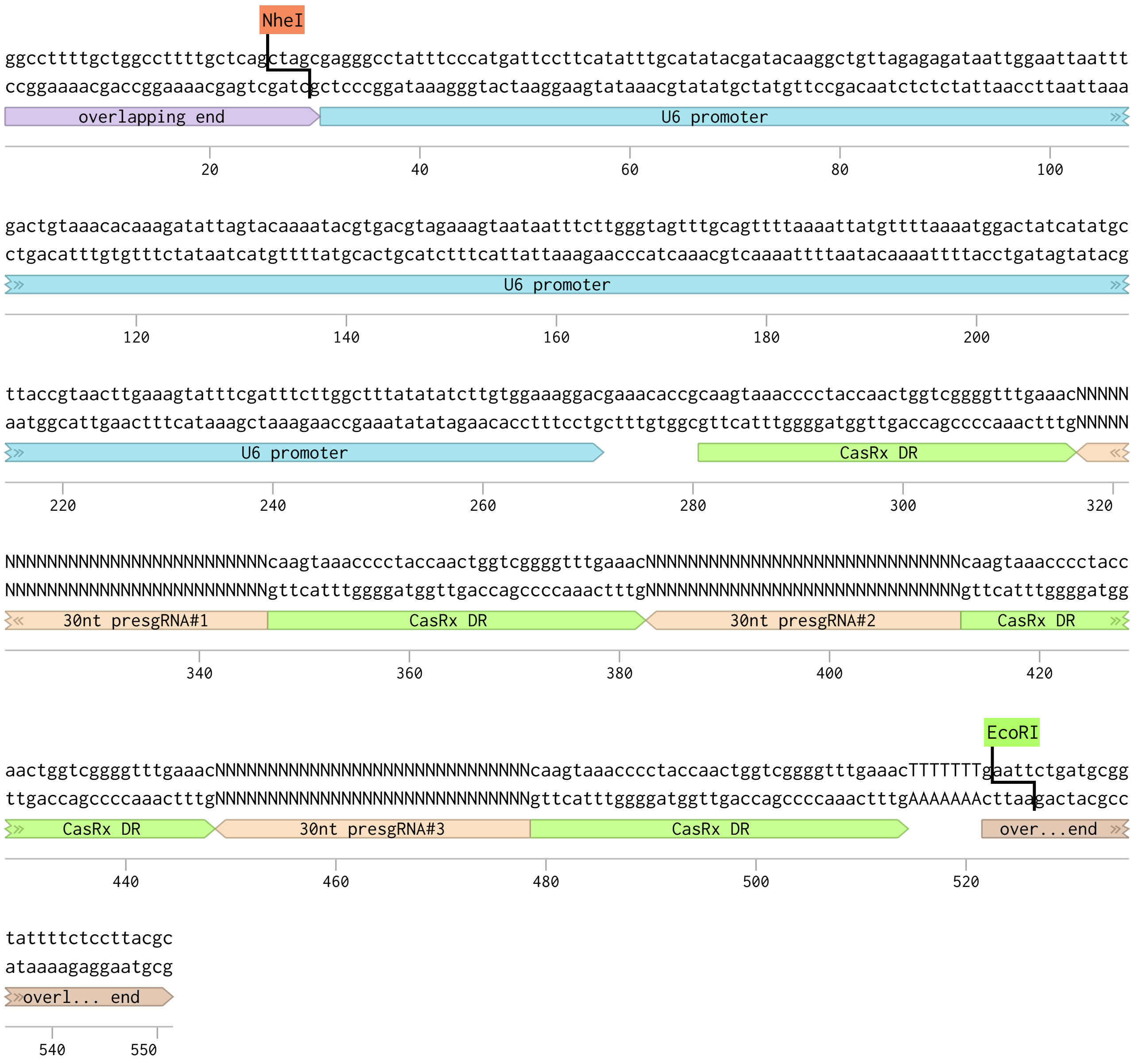

Figure S6. A circular map of control presgRNA plasmid. Created with Benchling.

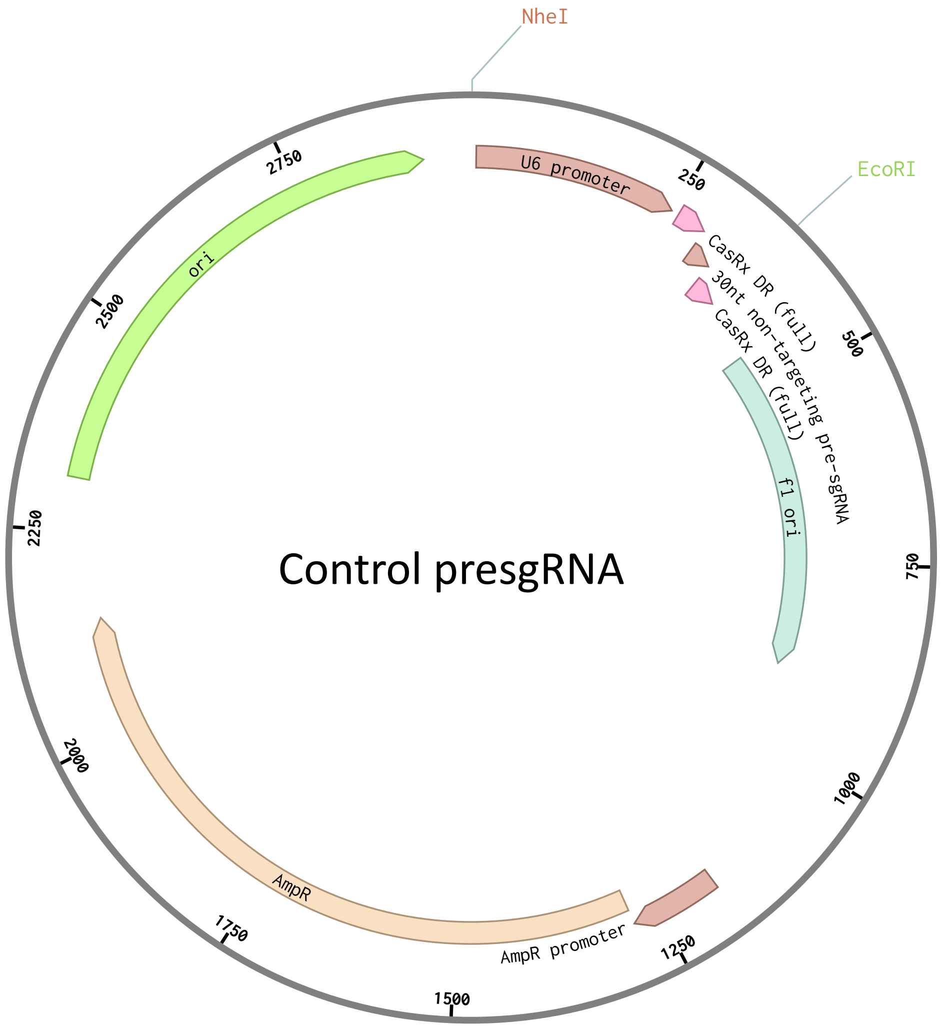

Figure S7. A circular map of VEGFA presgRNA plasmid. Created with Benchling.

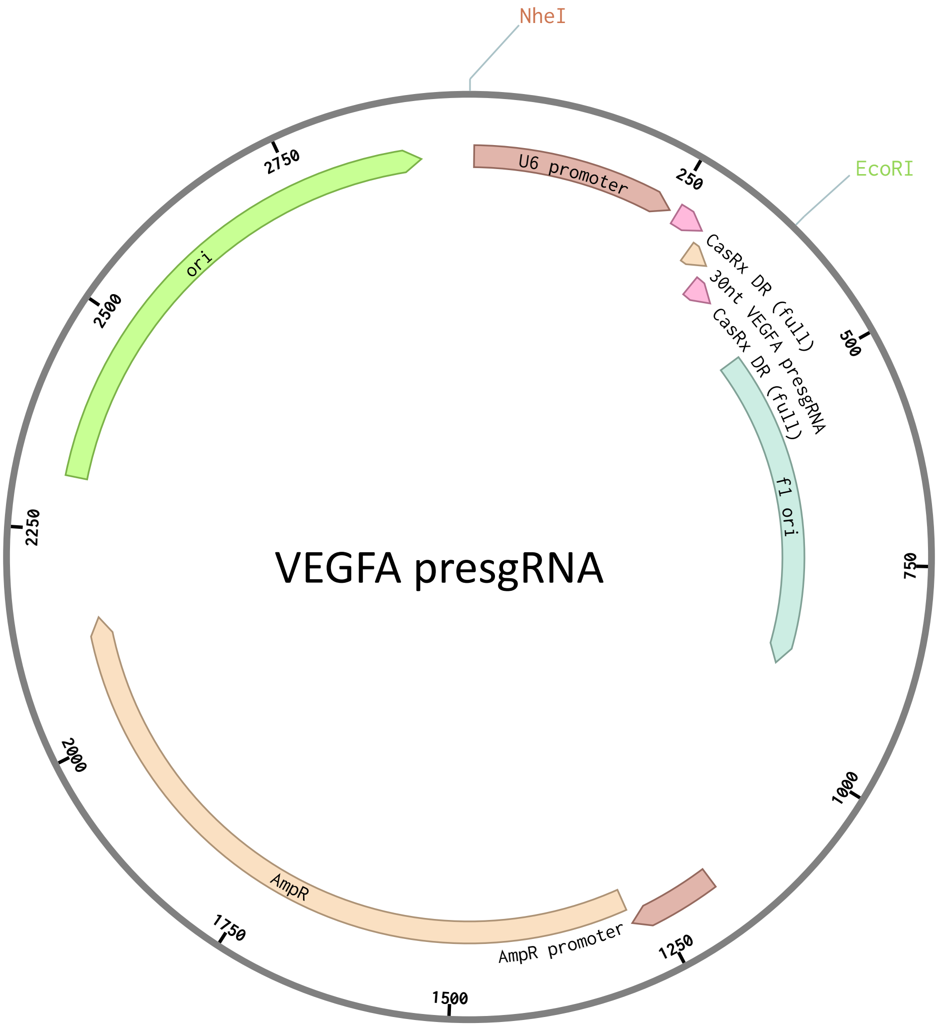

Figure S8. A circular map of pAAV-VEGFA presgRNA array plasmid. Created with Benchling.

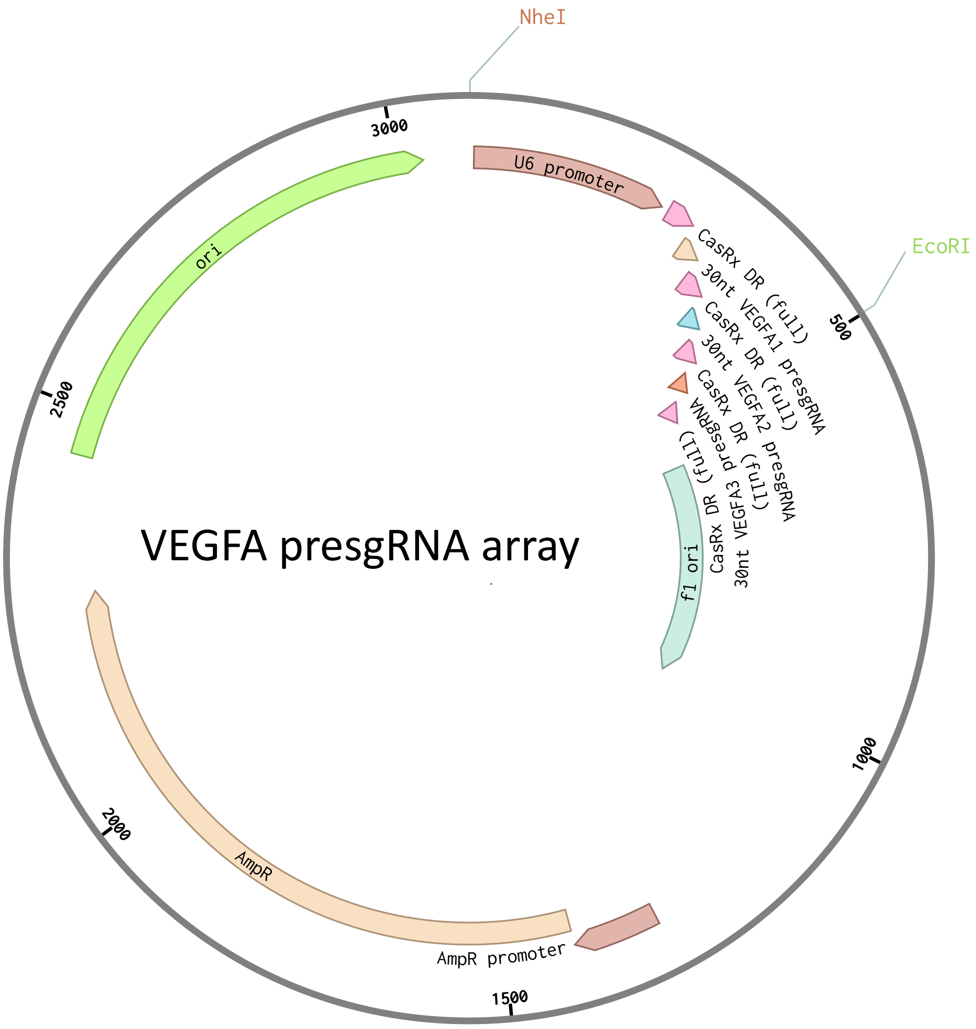

Figure S9. A circular map of pAAV-CasRx-control presgRNA plasmid. Created with Benchling.

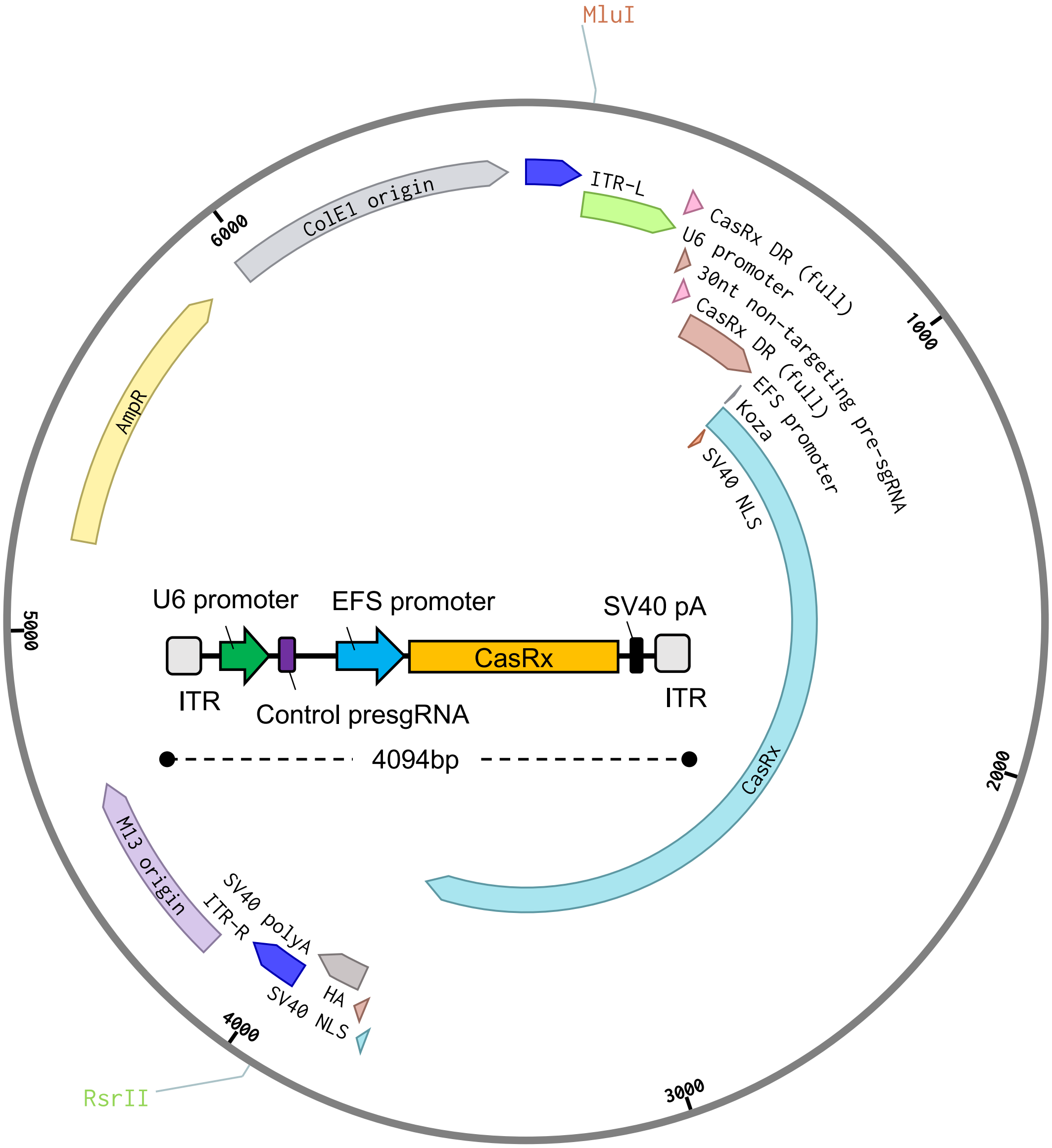

Figure S10. A circular map of pAAV-CasRx-VEGFA presgRNA plasmid. Created with Benchling.

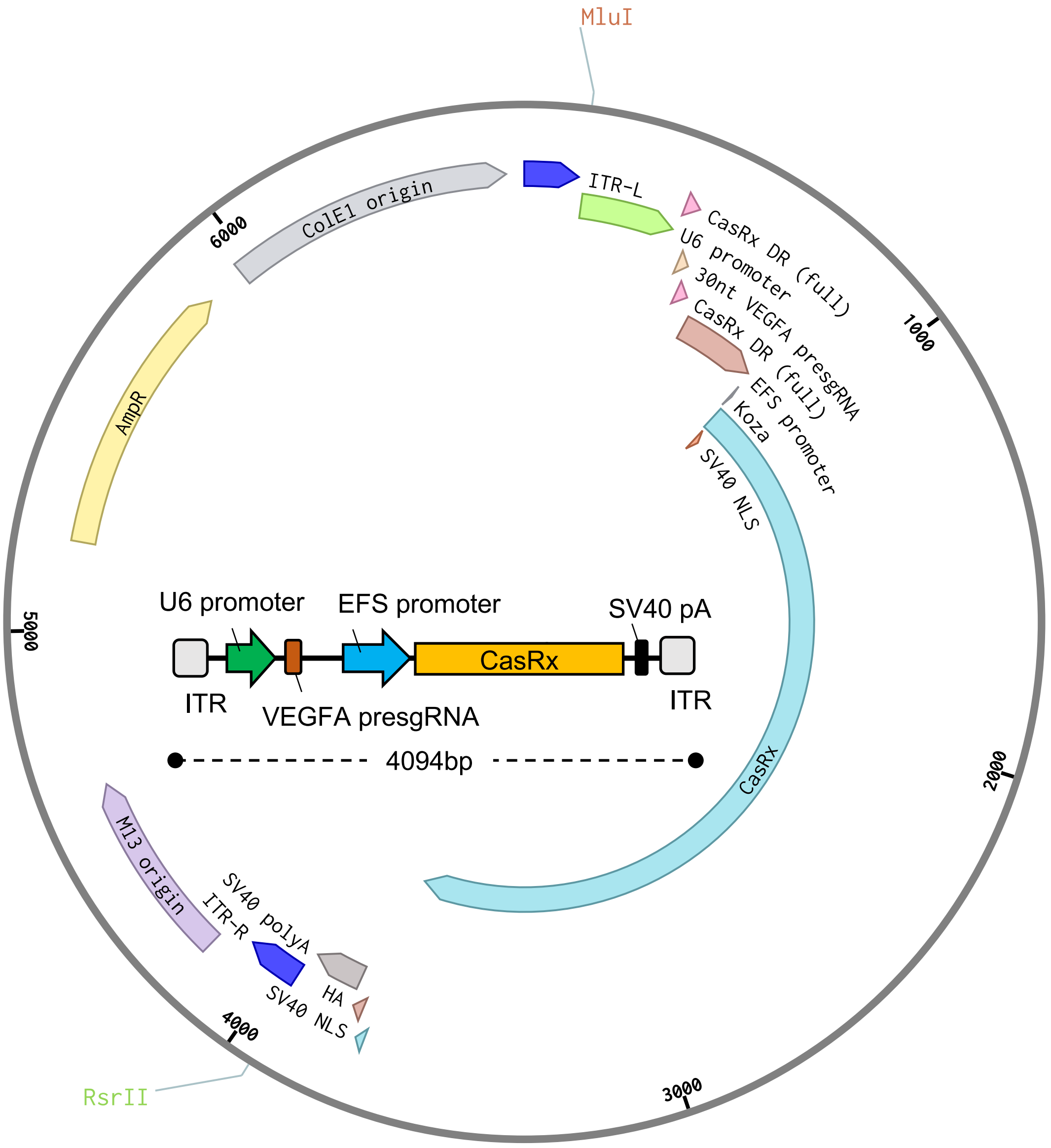

Figure S11. A circular map of pAAV-CasRx-VEGFA presgRNA array plasmid. Created with Benchling.

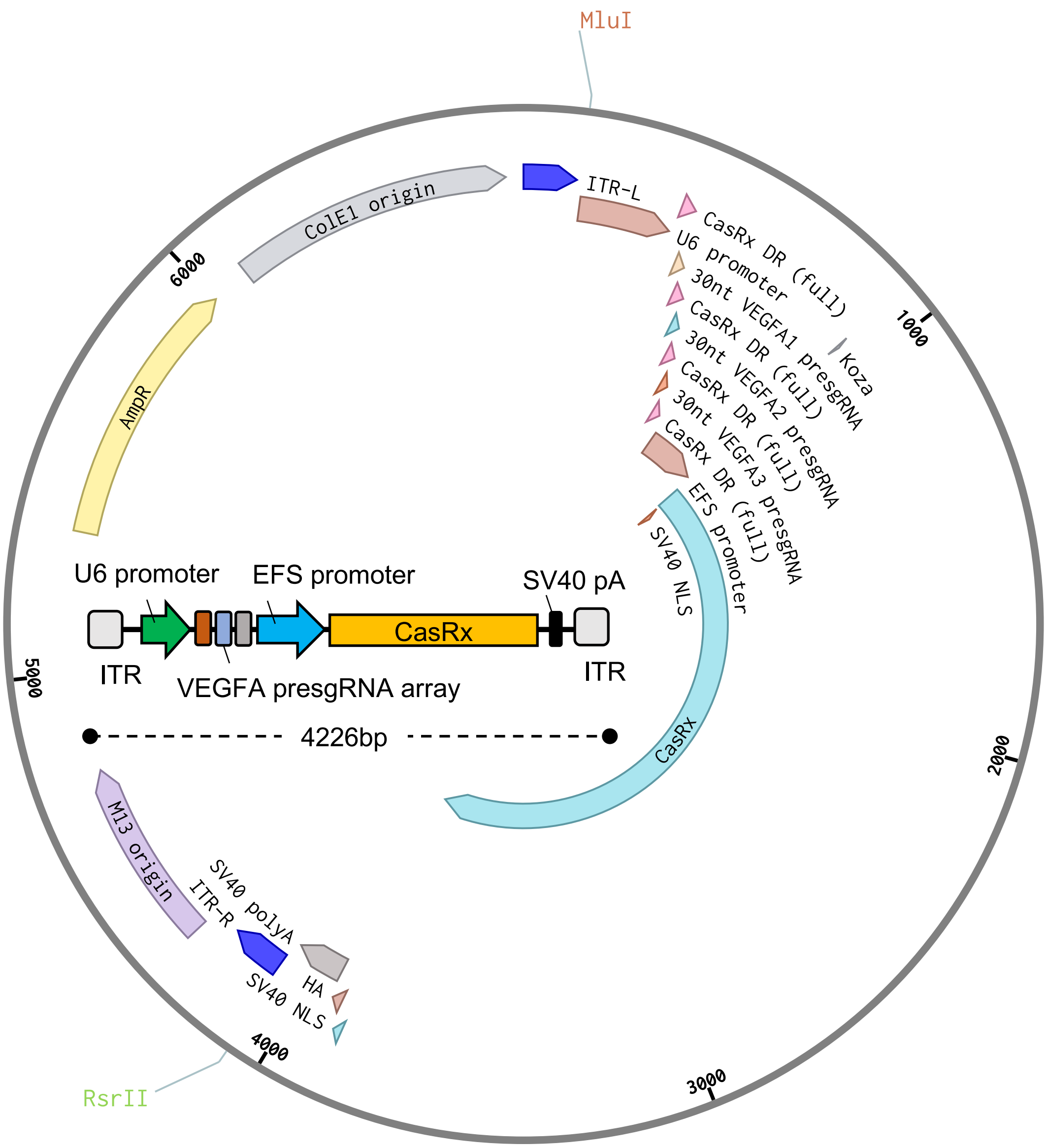

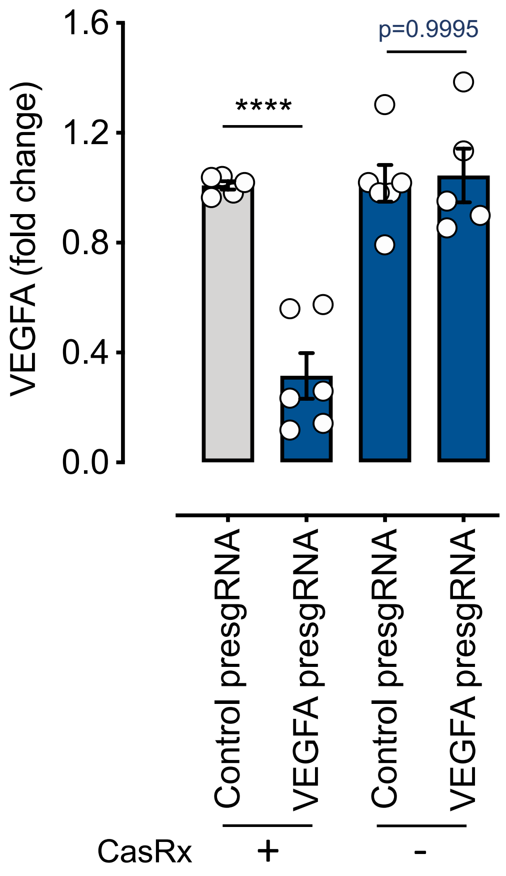

Figure S12. Expression of CasRx is essential for CRISPR-based RNA editing. HEK293FT cells were transfected with control (non-targeting) presgRNA, VEGFA presgRNA along with or without CasRx plasmid for 72 hours. The VEGFA mRNA level was determined by qPCR (n=5). Data are expressed as mean ± SEM. Statistical analysis was performed with GraphPad Prism 7 (GraphPad, San Diego, CA) and undertaken with One-way ANOVA and Tukey's multiple comparison test; ****P < 0.0001.

SUPPLEMENTARY METHODS

1. Step by step protocol for DNA Clean & Concentrator™-25 Kit (Zymo Research)

**Step 1**	Transfer DNA sample to 1.5 mL microcentrifuge tube and add 5 volumes of DNA Binding Buffer to each DNA sample. Vortex to mix thoroughly.
**Step 2**	Transfer mixture to Zymo-Spin Column in Collection Tube.
**Step 3**	Centrifuge at 12000 g for 30 seconds. Discard the flow-through.
**Step 4**	Add 200 µL DNA Wash Buffer to the column and centrifuge at 12000 g for 30 seconds.
**Step 5**	Repeat Step 4 once.
**Step 6**	Transfer column to new microcentrifuge tube and add 50 µL nuclease-free water to the column matrix and incubate for 3 minutes. Centrifuge at 12000 g for 30 seconds to elute the DNA.

2. Step by step protocol for QIAquick Gel Extraction Kit (Qiagen)

**Step 1**	Excise the DNA from the agarose gel with scalpel. Remove excess agarose to minimize gel size.
**Step 2**	Weigh the gel in colorless tube and add 3 volumes (µL) of Buffer QG to 1 volume (mg) of the gel.
**Step 3**	Incubate at 50 °C for 10 minutes. Vortex the tube every 2 minutes during the incubation.<br>**Note:** Make sure the gel is completely dissolved.
**Step 4**	Check whether the color of the mixture is yellow or not after the gel is completely dissolved.<br>**Note:** If the color is orange or violet, the pH of the mixture is higher than optimal pH (pH ≤7.5) that is essential for DNA absorption to the QIAquick membrane. Add 10 µL of 3 M sodium acetate, pH 5.0 and mix thoroughly to turn the mixture color to yellow.
**Step 5**	Add 1 volume (µL) of isopropanol to 1 gel volume (mg) of the sample and mix.
**Step 6**	Apply the sample to the QIAquick column on collection tube to bind DNA. Centrifuge at 12000 g for 1 minute. Discard the flow-through.

Step 7 Add 0.5 mL of Buffer QG to QIAquick column and centrifuge at 12000 g for 1 minute.

Step 8 Add 0.75 mL of Buffer PE to QIAquick column. Let stand for 3 minutes and centrifuge at 12000 g for 1 minute. Discard the flow-through.

Step 9 Centrifuge the QIAquick column at 12000 g for another 1 minute.

Step 10 Transfer QIAquick column to new microcentrifuge tube and add 50 μL of nuclease-free water to column matrix. Let stand for 3 minutes and centrifuge at maximum speed to elute DNA.

3. Step by step protocol for QIAprep Spin Miniprep Kit (Qiagen)

Step 1 Centrifuge 5 mL bacterial culture at 10000 rpm for 3 minutes at room temperature.

Step 2 Discard supernatant and resuspend cell pellet in 250 μL Buffer P1 and transfer to a microcentrifuge tube.

Step 3 Add 250 μL Buffer P2 and invert the tube 4–6 times to mix thoroughly. Lysis reaction time should not exceed 5 minutes.

Step 4 Add 350 μL Buffer N3 and immediately invert the tube 4–6 times to mix thoroughly.

Step 5 Centrifuge for 10 minutes at 13,000 rpm.

Step 6 Transfer the supernatant to the QIAprep spin column. Centrifuge at 13,000 rpm for 1 minute and discard the flow-through.

Step 7 Add 0.5 mL Buffer PB to wash the QIAprep spin column. Centrifuge at 13,000 rpm for 1 minute and discard the flow-through.

Step 8 Add 0.75 mL Buffer PE to wash the QIAprep spin column. Centrifuge at 13,000 rpm for 1 minute and discard the flow-through. Place the QIAprep spin column to new collection tube.

Step 9 Centrifuge at 13,000 rpm for 1 minute to remove residual wash buffer.

Step 10 Place the QIAprep column in new 1.5 mL microcentrifuge tube and add 50 μL nuclease-free water to the center of the QIAprep spin column to elute DNA. Let stand for 3 minutes, and centrifuge at 13,000 rpm for another 1 minute.

4. Protocol for QIAGEN® Plasmid Midiprep/Maxiprep Kit (Qiagen)

Step 1 Centrifuge bacterial culture at 6000 x *g* for 15 minutes at 4°C.

Step 2 Discard supernatant and resuspend cell pellet in 4 mL (for midiprep) or 10 mL (for maxiprep) Buffer P1.

Step 3 Add 4 mL (for midiprep) or 10 mL (for maxiprep) Buffer P2 and invert 4–6 times to mix thoroughly at room temperature. Lysis reaction time should not exceed 5 minutes.

Step 4 Add 4 mL (for midiprep) or 10 mL (for maxiprep) prechilled Buffer P3 and invert 4–6 times to mix thoroughly. Incubate on ice for 15 minutes (for midiprep) or 20 minutes (for maxiprep).

Step 5 Centrifuge at 20,500 x *g* for 30 minutes at 4°C.

Step 6 Add 4 mL (for midiprep) or 10 mL (for maxiprep) Buffer QBT to QIAGEN-tip for equilibration. Empty the column by gravity flow.

Step 7 Apply the supernatant from step 5 to the QIAGEN-tip and allow it to move through the QIAGEN-tip by gravity flow.

Step 8 Add 2 x 10 mL (for midiprep) or 2 x 30 mL (for maxiprep) Buffer QC to wash the membrane. Allow itto move through the QIAGEN-tip by gravity flow.

Step 9 Elute DNA with 5 mL (for midiprep) or 15 mL (for maxiprep) Buffer QF into new 15 mL (for midiprep) or 50 mL (for maxiprep) centrifuge tubes.

Step 10 Then, used PureLink™ HiPure Precipitator Module to precipitate DNA. Add 3.5 mL (for midiprep) or 10.5 mL (for maxiprep) isopropanol to eluted DNA and inverse the tube to mix thoroughly. Incubate for 2 minutes.

Step 11 Screw the precipitator on 30 mL syringe and transfer the liquid into the syringe.

Step 12 Insert the plunger to push out the liquid and then air dry twice.

Step 13 Add 5 mL of 70 % ethanol to wash.

Step 14 Repeat step 13.

Step 15 Change the precipitator from 30 mL syringe to 5 mL syringe.

Step 16 Add 0.75 mL (for midiprep) or 1 mL (for maxiprep) TE buffer into the syringe, place the syringe into microcentrifuge tube and push out the liquid very slowly to collect eluted DNA.

Note: Push out the liquid as slowly as possible to get higher DNA concentration.

Step 17 Transfer eluted DNA back to the syringe and elute again.

5. Step by step protocol for Quick-RNA® Miniprep Kit (Zymo Research)

Step 1 Remove media from plate wells. Add 300 µl RNA Lysis Buffer to each well.

Step 2 Remove cells from each well by pipetting.

Step 3 Collect sample to microcentrifuge tube.

Step 4 Add 1 volume of 100 % ethanol to each sample and mix well.

Step 5 Transfer the sample into Zymo-Spin™ IIICG Column in a collection tube and centrifuge at 12000 g for 1 minute. Discard the flow-through.

Step 6 Add 400 µL RNA Prep Buffer to the column and centrifuge at 12000 g for 1 minute. Discard the flow-through.

Step 7 Add 700 µL RNA Wash Buffer to the column and centrifuge at 12000 g for 1 minute. Discard the flow-through.

Step 8 Move column to new collection tube.

Step 9 Add 400 µL RNA Wash Buffer and centrifuge at 12000 g for 2 minutes. Discard the flow-through.

Step 10 Centrifuge the column at 12000 g for another 1 minute to remove residual wash buffer.

Step 11 Carefully transfer the column into new microcentrifuge tube.

Step 12 Add 50 µL nuclease-free water to the column matrix. Let stand for 3 minutes, and centrifuge at 12000 g for 1 minutes.
